# A genome-wide association meta-analysis of cholesterol synthesis intermediates identifies three associations for lanosterol

**DOI:** 10.1016/j.ebiom.2026.106144

**Published:** 2026-01-30

**Authors:** Franz Förster, Katrin Horn, Janne Pott, Graciela E. Delgado, Marcus E. Kleber, Winfried März, Angela Patricia Moissl-Blanke, Günther Silbernagel, Melanie Waldenberger, Harald Grallert, Annette Peters, Christian Gieger, Ronny Baber, Holger Kirsten, Markus Loeffler, Berend Isermann, Joachim Thiery, Peter Kovacs, Anke Tönjes, Michael Stumvoll, Helena Gylling, Mika Kähönen, Terho Lehtimäki, Pashupati Prasad Mishra, Olli Raitakari, Uta Ceglarek, Markus Scholz

**Affiliations:** a Institute for Medical Informatics, Statistics and Epidemiology (IMISE), Medical Faculty, Leipzig University, Leipzig, Saxony, 04107, Germany; b IMB gGmbH, Mainz, Rhineland-Palatinate, 55128, Germany; c MRC Biostatistics Unit, University of Cambridge, Cambridge, CB2 0SR, United Kingdom; d LURIC Study GmbH, Aystetten, D-86482, Germany; e Department of Medicine I (Cardiology, Angiology, Hemostaseology, Intensive Care), Medical Faculty Mannheim, University of Heidelberg, Mannheim, 68167, Germany; f SYNLAB MVZ Humangenetik Mannheim, Mannheim, 68163, Germany; g Department of Medicine III (Cardiology, Pneumonology, Angiology), Medical Faculty Heidelberg, University of Heidelberg, Heidelberg, 69117, Germany; h Synlab Academy, Synlab Holding Deutschland GmbH, Augsburg, 86156, Germany; i Clinical Institute of Medical and Chemical Laboratory Diagnostics, Medical University Graz, Graz, 8010, Austria; j Division of Angiology, Department of Internal Medicine, Medical University of Graz, Graz, 8010, Austria; k Department of Angiology, Kantonsspital St. Gallen, HOCH Health Ostschweiz, St. Gallen, 9000, Switzerland; l Research Unit Molecular Epidemiology, Helmholtz Zentrum München, German Research Center for Environmental Health (GmbH), Neuherberg, D-85764, Germany; m Institute of Epidemiology, Helmholtz Zentrum München, German Research Center for Environmental Health (GmbH), Neuherberg, D-85764, Germany; n German Centre for Cardiovascular Research (DZHK), Partner Site Munich Heart Alliance, Munich, Germany; o German Center for Diabetes Research (DZD), Neuherberg, D-85764, Germany; p Institute for Medical Information Processing, Biometry, and Epidemiology (IBE), Pettenkofer School of Public Health, Faculty of Medicine, Ludwig Maximilians University, Munich, 81377, Germany; q LIFE – Leipziger Forschungszentrum für Zivilisationserkrankungen, Leipzig, Saxony, 04103, Germany; r Institute for Laboratory Medicine Clinical Chemistry and Molecular Diagnostics, University Hospital Leipzig, Leipzig, Saxony, 04103, Germany; s Medical Faculty, Christian-Albrechts-Universität zu Kiel, Kiel, 24118, Germany; t Medical Department III – Endocrinology, Nephrology, Rheumatology, University Hospital Leipzig, Leipzig, 04103, Germany; u Heart and Lung Center, Cardiology, University of Helsinki and Helsinki Univ. Hospital, Helsinki, 00290, Finland; v Department of Clinical Physiology, Tampere University Hospital, and Finnish Cardiovascular Research Center - Tampere, Faculty of Medicine and Health Technology, Tampere University, Tampere, 33521, Finland; w Department of Clinical Chemistry, Fimlab Laboratories, and Finnish Cardiovascular Research Center - Tampere, Faculty of Medicine and Health Technology, Tampere, 33521, Finland; x Centre for Population Health Research, University of Turku and Turku University Hospital; Research Centre of Applied and Preventive Cardiovascular Medicine, University of Turku, Turku, Finland; y Department of Clinical Physiology and Nuclear Medicine, Turku University Hospital, Turku, 20520, Finland

**Keywords:** GWAS, Cholesterol biosynthesis, Lanosterol, Desmosterol, Coronary artery disease

## Abstract

**Background:**

Cholesterol is a main contributor to coronary artery disease (CAD). Although the genetic basis of blood cholesterol concentration is well studied, there is currently a lack of studies investigating the genetics of its precursors from *de novo* biosynthesis.

**Methods:**

We conducted a genome-wide association meta-analysis, combining data from KORA, LIFE-Heart, LIFE-Adult, LURIC, the Sorbs study, and YFS, resulting in up to 10,519 individuals. We investigated 14 traits related to serum concentrations of lanosterol, desmosterol, and cholesterol. Direct and indirect effects of lanosterol on CAD were investigated with a Mendelian randomisation mediation analysis.

**Findings:**

Our analysis revealed four genome-wide significant (p < 5 × 10^−8^) associations not previously reported in the GWAS catalogue. These include two loci without prior connection to cholesterol, associated with lanosterol (7q21.2, *CYP51A1*) and free cholesterol (11q14.1), and two associations with lanosterol at loci previously reported for cholesterol (5q13.3, *HMGCR;* 11q23.3, APO cluster). We also replicated eight loci previously reported for associations with cholesterol-related traits. Lanosterol exhibited significant total and indirect effects on CAD, but its direct effect was not significant.

**Interpretation:**

We demonstrate that the investigation of intermediate phenotypes can help to functionally fine map previously reported associations for cholesterol, improving our understanding of genetic regulation of cholesterol concentrations. Further, the effect of lanosterol on CAD is probably fully mediated by total cholesterol.

**Funding:**

This investigation was primarily funded by the ministry for science and health of the Rhineland-Palatinate through the CoAGE graduate programme. A complete list of funding organisations is provided in the acknowledgements.

**Research in context**

**Evidence before this study**

Cholesterol concentrations in blood are an established risk factor for coronary artery disease. Consequentially, it has been a major focus to investigate the genetic influence on cholesterol levels in various lipid species, with the GWAS catalogue listing 437 studies and 9923 associations for total cholesterol alone (as of August 8th, 2025). This also enabled large genome-wide association meta-analyses, like the Global Lipids Genetics Consortium, which investigated 1.6 million individuals. In contrast, genetic investigations of cholesterol precursors, like lanosterol and desmosterol, are limited by low sample size. A genome-wide association study was conducted in 300 pigs to investigate lanosterol, lathosterol, desmosterol, in addition to several phytosterol and cholesterol traits, reporting a single association for desmosterol. In humans, only few investigations have been performed. A genome-wide association analysis based on the studies EUGENE2 and METSIM did not report genome-wide significant associations for lathosterol or desmosterol. Similarly, a genome-wide association study of sterols in 40 individuals of the GOLDN study did not find genome-wide significant findings for lanosterol or desmosterol. Consequently, the GWAS catalogue lists only a single association for lanosterol (reported trait: free brassicasterol to free lanosterol) and no known associations for desmosterol (as of August 8th, 2025).

**Added value of this study**

In this study we perform a genome-wide association meta-analysis to study the genetic basis of lanosterol, desmosterol, and cholesterol. Through extensive bioinformatic annotation and comparison of association patterns, we elucidate potential underlying mechanisms of observed associations.

**Implications of all the available evidence**

Our findings imply that two associations previously reported for cholesterol are caused by changes in lanosterol concentrations. This helps to increase our understanding of the genetic influence on cholesterol synthesis and, by extension, the genetic predisposition of coronary artery disease. It also demonstrates the value of investigating a broad range of cholesterol precursors in future studies.

## Introduction

Cholesterol is a major risk factor for atherosclerotic cardiovascular disease,^1^ which includes coronary artery disease (CAD), the leading cause of death worldwide.^2^ The investigation of cholesterol homoeostasis has therefore become an area of strong medical interest. Although extensive research has been performed to elucidate the genetic basis of blood cholesterol concentrations,^3^ biosynthetic precursors of cholesterol have rarely been investigated since standard laboratory assessments performed in large population studies typically do not cover these lipid species, due to analytical limitations.

The zoosterol cholesterol is an essential compound of the cell membrane, regulating fluidity, permeability and compartmentalisation. It also is the backbone for the synthesis of steroid hormones, such as oestrogen or testosterone, as well as vitamin D analogues.^4^ Cholesterol homoeostasis is the result of balancing uptake, efflux, *de novo* synthesis, and esterification.^5^ The majority of cholesterol stems from *de novo* synthesis (700–900 mg/day), while dietary absorption is a secondary source (300–500 mg/day).^6^ Cholesterol uptake can be estimated by measuring serum levels of plant sterols, which are exclusively derived from diet. Assessment of *de novo* cholesterol synthesis can be done by measuring the cholesterol precursors lanosterol and desmosterol, for which few nutritional sources are available.^7^ The synthesis primarily takes place in the liver, accounting for 50% of newly synthesised sterols in rats,^8^ and can be performed through two major pathways. Starting from lanosterol, the Bloch pathway uses desmosterol as an intermediate,^9^ whereas the Kandutsch-Russell pathway synthesises cholesterol via lathosterol and 7-dehydrocholesterol.^10^ In practice, these pathways are often combined and subject to a complex regulatory network (for an overview, see Luo et al.^11^).^12^

For transport in blood, cholesterol is 75% esterified as part of plasma lipoproteins, serving as shuttles towards peripheral tissues, whereas lanosterol is transported unesterified. During transportation, cholesterol can enter vascular walls, contributing to arterial plaque formation and increasing the risk for CAD.^4^ The prevalence of coronary heart disease is two to five times higher in men than in women, which can partially be attributed to sex-differences in cholesterol profiles.^13^ For example, atherogenic low-density lipoprotein (LDL) concentrations are lower in women below the age of 50 while high-density lipoprotein (HDL) concentrations, supporting reverse cholesterol transport, are increased in women of all ages.^14^ These sex-differences potentially extend to cholesterol metabolism with hepatic gene-expression analysis in hypercholesterolemic mice revealing 1390 genes with differential expression between chromosomal sexes, including genes related to fatty acid metabolism.^15^ Further, oestrogen affects bile acid metabolism, potentially implicating effects on cholesterol homoeostasis.^16^

Extensive research has been performed with regard to the genetic basis of lipid traits, with the Global Lipids Genetics Consortium investigating 1.6 million individuals.^3^ However, previous studies focused on cholesterol concentrations in lipoproteins such as HDL-cholesterol and LDL-cholesterol (LDL-C) rather than chemical precursors of cholesterol. The investigation of such intermediate phenotypes promises to improve our understanding of the genetic regulation of cholesterol homoeostasis. Cholesterol levels in blood are influenced by complex interrelated mechanisms, including transport, *de novo* synthesis, excretion, and storage. In contrast, intermediate phenotypes can more closely reflect actual biosynthetic processes and their genetic regulations, especially if biological relevance is pathway specific. The potential reduction in environmental effects on these chemical processes could also result in increased power for finding new genetic associations. Furthermore, the use of intermediate phenotypes allows better biological interpretability of identified associations, because intermediate compounds represent specific steps in the biosynthesis pathway. These ideas motivated an investigation of the genetic basis of lanosterol, lathosterol, desmosterol, in addition to several phytosterol and cholesterol traits in 300 pigs.^17^ The study identified a single genome-wide significant locus for a cholesterol precursor, namely desmosterol, near the *DHCR24* gene. Further, several sterols, including lanosterol and desmosterol, were used in a genome-wide association study (GWAS) of 40 individuals, but genome-wide significant findings were restricted to β-sitosterol, 7a-hydroxycholesterol, and campesterol.^18^ Another GWAS, investigating sterol levels in the studies EUGENE2 (n = 273) and METSIM (n = 1050), included lathosterol and desmosterol, but did not report genome-wide significant associations for these traits.^19^

In this study, we investigate intermediate phenotypes of cholesterol biosynthesis in humans. For this purpose, we combine six studies in a genome-wide association meta-analyses (GWAMA) of sterol traits and investigate concentrations of lanosterol, desmosterol and cholesterol, including non-esterified and esterified species, as well as ratios representing reaction equilibria. We aim to elucidate biological relevance of associated variants by bioinformatic annotation and colocalisation analyses with expression quantitative trait loci (eQTLs) and CAD. We also performed sex-stratified analyses to detect genetic sex interactions.

## Methods

### Participating studies

This meta-analysis combined data from six independent studies: the Ludwigshafen Risk and Cardiovascular Health (LURIC) study,^20^ Cooperative Health Research in the Region of Augsburg (KORA),^21^^,^^22^ LIFE-Heart,^23^ LIFE-Adult,^24^ Sorbs,^25^^,^^26^ and the Young Finns study (YFS).^27^ All participants were of European ancestry. A detailed study description and overview of study characteristics is given in Supplementary Tables S1 and S2.

### Zoosterol measurement and phenotype definition

Serum concentrations for lanosterol (non-esterified/free), desmosterol (free, esterified, and total) and cholesterol (free, esterified, and total) were available in all or a subset of participating studies (see Supplementary Table S3). Measurements in KORA, LIFE-Adult, LIFE-Heart, and Sorbs were performed with liquid-chromatography tandem mass-spectrometry at the Institute of Laboratory Medicine, Leipzig University, according to the protocol described in Lembcke et al.^28^ Batch-effect correction of these measurements was performed with the R-package ‘sva’ (function ‘ComBat’).^29^ In LURIC and YFS, sterols were measured with gas chromatography-mass spectrometry. A detailed protocol of sterol measurements in LURIC is described in Silbernagel et al.^30^

From the measured zoosterol concentrations, we calculated the following ratios, representing biologically relevant reaction equilibria: free to esterified cholesterol (representing activity of lecithin–cholesterol acyltransferase), free to esterified desmosterol, free desmosterol to free cholesterol, total desmosterol to total cholesterol, free lanosterol to total cholesterol, free desmosterol to free lanosterol, and total desmosterol to free lanosterol. Thus, a total of 14 traits were analysed.

### Clustering of sterol traits

The correlation structure of zoosterol traits was investigated with hierarchical clustering. We used individual level data from KORA, LIFE-Heart, LIFE-Adult, and Sorbs, as these studies used the same sterol measurement technique and because individual level data were available. Pairwise partial correlations controlling for age, sex, log-transformed body mass index (BMI), diabetes status, lipid lowering medication, and study was used as distance measure between log-transformed sterol traits. The function ‘pcor.test’ included in the package ‘ppcor’ (v. 1.1)^31^ with Pearson regression was used for this purpose. Coefficients of correlation are shown in Supplementary Figure S1. Ward's hierarchical clustering was performed as implemented in the R function ‘hclust’ included in the stats package (v. 4.5.1),^32^ using $1-\left|r_{ij}\right|$ for the initial distance matrix.

### Genotyping and imputation

Genotyping was performed by various microarray platforms: Illumina Omni 2.5/Illumina Omni Express (KORA), Affymetrix Axiom CEU1 (LIFE-Adult, LIFE-Heart), Affymetrix custom array (LIFE-Heart), Affymetrix Gene Chip Human Mapping 500 K Array Set (Sorbs), Affymetrix Genome-Wide Human SNP Array 6.0 (LURIC, Sorbs), Illumina 200 k MetaboChip (LURIC), and Illumina Human 670 k BeadChip (YFS). Study-wise quality control (QC) was performed according to the procedures described in Supplementary Table S2.

IMPUTE2^33^ was used for genotype imputation with 1000 Genomes Phase 1^34^ (LURIC, LIFE-Heart, LIFE-Adult, Sorbs, YFS) and 1000 Genomes Phase 3^35^^,^^36^ (KORA) as references. Conversion to the forward strand was performed using hg19 coordinates.

### Single study genome-wide association analysis

In each cohort, a GWAS was performed according to a standardised analysis plan. KORA, LIFE-Heart, LIFE-Adult, and Sorbs were analysed centrally at the IMISE, Leipzig University, while LURIC and YFS were analysed in the respective study centre. Log-transformation was applied to the traits prior to analysis to approximate a Gaussian distribution. In each study, regression analysis was performed with an additive gene model, adjusted for age, genetically verified sex, log(BMI), diabetes status, and lipid lowering medication (i.e. Anatomical Therapeutic Chemical codes starting with ‘C10’). Additionally, LURIC adjusted for the first three principal components and traits in the Sorbs study were corrected for relatedness structure with the function ‘polygenic’ from the package ‘GenABEL’ (v. 1.8).^37^ Association analyses were performed with PLINK 1.9 (LIFE-Adult, LIFE-Heart, Sorbs), Plink 2.0 (LURIC, KORA),^38^ and SNPTEST 2.5 (YFS).^39^ Complete X-inactivation was assumed for the analysis of X-chromosomal markers.

Harmonisation of summary statistics of all studies was performed using EasyQC (v. 9.2)^40^ and custom scripts. We discarded variants not in the reference panel (1000 Genomes phase 1, version 3, European ancestry), with missing values in statistics (e.g. beta estimates, imputation quality score), missing allele information (effect allele, other allele, effect allele frequency), and mismatching alleles or chromosomal position with respect to the reference. Genetic variants were filtered for minor allele frequency (MAF) > 1%. Genotyped variants were filtered for call rate > 95% and Hardy–Weinberg equilibrium (p > 10^−6^), while imputed variants were filtered for imputation quality score > 0.5 and for deviation from reference allele frequency < 20%. Finally, alleles were harmonised such that the effect allele was consistent across studies. Genomic control was applied if the variance inflation factor λ was > 1.

### Meta-analysis and post-meta quality control

We performed fixed-effect meta-analysis with a custom script, weighting study effects by their inverse variance. Post meta-analysis, we discarded variants only present in one study, with a sample-size weighted MAF ≤ 1%, a sample-size weighted imputation quality-score ≤ 0.7 or heterogeneity I^2^ ≥ 0.85.

### Statistics

The p-value for the association of a variant in the meta-analysis was calculated with a two-sided Z-test. No determination of sample size, randomisation, blinding, or definition of inclusion and exclusion criteria was performed.

### Heritability and genetic correlation

Heritability of sterol traits was estimated with linkage disequilibrium (LD)-score regression as implemented in LDSC (v. 1.0.1).^41^ Variants were quality filtered according to the default QC criteria of the tool. The LD scores for European populations of 1000 Genomes phase 3 provided by the authors of the package were used as reference. We tested for significant heritability (p < 0.05) in a one-sided test.

We further investigated genetic correlation between zoosterols. We limited our investigation to traits with mean X^2^ > 1.02, as recommended by the authors of the tool, and significant heritability. Quality control and references for the genetic correlation analysis were identical to the heritability analysis. We considered genetic correlation to be significant when p < 0.05 in a two-sided test.

### Locus definition

Variants with p < 5 × 10^−8^ were considered genome-wide significant. Genomic loci containing genome-wide significant variants were defined across all 14 traits simultaneously. We defined loci as the 1 Mb region around the variant with the lowest p-value across all association analyses (index variant) and iterated until all genome-wide significant associations were assigned to a locus. Overlapping loci were merged, keeping the variant with the lowest p-value as index variant. The trait where the lowest p-value of a locus was observed was considered the best-associated phenotype of the respective locus. To reduce the risk of false positive associations, loci where no other variant besides the index variant reached at least suggestive significance (p < 10^−6^) were removed.

### Influence of analysis platform

To check potential confounding due to different sterol measurement procedures between studies, we performed meta-regression for all index variants, including the zoosterol measurement procedure (liquid-chromatography tandem mass-spectrometry or gas chromatography-mass spectrometry) as a binary moderator variable and treating liquid-chromatography tandem mass-spectrometry as the reference group. The function ‘rma’ from the R package ‘metafor’ (v. 4.8-0) was used for this purpose.^42^ Significant moderator effects were assumed if the false discovery rate (FDR) corrected p-values of the omnibus test for moderator effects were significant (p < 0.05). Further, we compared results from fixed effect and random effects model for index variants showing moderate heterogeneity (I^2^ > 0.4) in their respective best associated phenotype, testing for significant differences with a two-sided two-sample Z-test (p < 0.05).

### Influence of lipid-lowering medication

The influence of including lipid lowering medication as a binary covariate was assessed through a sensitivity analysis based on KORA, LIFE-Heart, LIFE-Adult, and the Sorbs study. For these four studies, GWAS and meta-analysis were performed as described in previous sections, using Plink 2.0.^38^ In the first setting, all individuals were included and adjustment for lipid treatment was performed as for the primary analysis by introducing a respective binary covariate into the regression model. In the second setting, all individuals with lipid-lowering medication were excluded from the analysis. Pearson correlation of effect estimates between the two analysis settings was calculated for all variants fulfilling the QC criteria described above and having the same number of studies present in both settings. Further, correlation was calculated for the subset of filtered variants showing suggestive significance (p < 10^−6^) in either of the two settings. For index variants, we searched for significant differences of effect estimates with a two-sided two-sample Z-test (p < 0.05).

### Conditional analysis

Conditional and joint (COJO) analysis was performed to identify secondary associations, i.e. genome-wide significant variants that are independent of the respective index variant. For this purpose, we employed the function ‘cojo-slct’ from ‘GCTA-COJO’ (v. 1.92.0 beta 2).^43^^,^^44^ This analysis was performed for all loci and all traits associated with the respective locus. In case of multiple independent variants, conditional statistics were calculated by conditioning each variant on the other independent variants, respectively. For this we applied the function ‘cojo-cond’ from the GCTA-COJO package.^43^^,^^44^ The LD-reference for COJO was established by combining genotypes from LIFE-Heart and LIFE-Adult.

### Credible set definition and annotation

99% credible sets (CSs) for independent variants were determined by calculating approximate Bayes factors (ABFs) for variants at the locus using the Wakefield method^45^ implemented in the R-package ‘gtx’ (v. 0.0.8).^46^ The difference between the 97.5% and the 2.5% percentile of the effect size distribution for the respective locus was used for prior estimation. The posterior probability (PP) for each variant was determined by dividing its ABF by the sum of the ABFs of all variants at the locus. Variants were added to the CS in order of decreasing PP until the cumulative PP of variants within the CS reached 99%. We used association statistics from the respective best-associated phenotype. Conditional statistics were used when multiple independent variants were identified for a single locus. In case of large CSs (n ≥ 100), we primarily focussed the evaluation on variants with a sufficiently high probability of being causal (PP > 1%).

Bioinformatic annotation of variants included in 99% CSs was performed with a custom pipeline and various resources. Variants were annotated by Ensembl 2018^47^ based gene look-up in a region of ±250 kb around the variant, combined annotation dependent depletion (CADD) score,^48^ LD (r^2^ > 0.3) with other associated variants according to the GWAS catalogue (downloaded on November 13th, 2024),^49^ and LD with eQTLs of the GTEx V8 catalogue (dbGaP Accession phs000424.v8.p2, downloaded on June 10th, 2020),^50^ as well as in-house eQTL references. As LD reference, we used the 1000 Genomes Phase 1 version 3 panel for European populations.^34^ We considered associations to be previously unreported with regard to the GWAS catalogue, when the index variant was not in LD (r^2^ > 0.3) with previously reported variants for the respective trait. In the following, we will use the term novel associations as a shorthand for such associations. We considered variants with CADD ≥ 10 as high CADD variants, implying high biological relevance.

Candidate genes were assigned through biologically informed manual annotation. All genes in a ±250 kb window around variants within the CS were extracted and the respective biological function retrieved from GeneCards (v. 5.19).^51^^,^^52^ If protein-coding genes with known functional relation to sterol metabolism were found, these were selected as candidates. Otherwise, we selected genes where such a relation could reasonably be established. In case of multiple plausible candidate genes, we prioritised the gene closest to the index variant.

### Colocalisation analysis

To test for shared genetic signals of zoosterols and other traits, colocalisation analysis was performed with the function ‘coloc.abf’ from the package ‘coloc’ (v. 5.2.3).^53^ Conditional statistics for zoosterols were used if a locus contained multiple independent variants. We assumed colocalisation if the posterior probability for colocalisation PP(H4) was ≥ 80%. We utilised colocalisation analysis to verify identity of signals for loci with multiple associated zoosterol traits. We further tested for colocalisation of respective best-associated traits with CAD. Summary statistics for CAD were obtained from Aragam et al. (downloaded on March 11th, 2025).^54^

To identify possible candidate genes, we analysed colocalisation between genome-wide significant signals of zoosterols and cis-eQTLs of selected genes in the following tissues: whole blood, the measurement site of zoosterol concentrations in our study, liver, the primary organ of cholesterol synthesis, subcutaneous adipose tissue, the major cholesterol storage site, adrenal gland, testis and ovary, sites of steroid hormone synthesis, small intestine (terminal ileum), the primary site of cholesterol intake, and skeletal muscle, involved in the regulation of cholesterol homoeostasis. For each locus, we selected genes in a ±250 kb window around variants of the respective CS as well as genes affected by cis-eQTLs in LD (r^2^ > 0.3) with these variants (see section ‘Credible set definition and annotation’). For these genes, eQTL data was extracted for the respective locus from GTEx V8 (dbGaP Accession phs000424.v8.p2, downloaded on June 10th, 2020).^50^ We did not investigate trans-eQTLs due to their higher risk of being false positive.

### Sex interaction analysis

We analysed index variants for single-nucleotide polymorphism (SNP)-by-sex interaction. For this purpose, sex-stratified GWAMA was performed including four studies (KORA, LIFE-Heart, LIFE-Adult, Sorbs) for which individual-level genotype data were accessible. Single-study genome-wide association analysis (GWAS), QC, and meta-regression were performed as described for the main analysis, but without including sex as a covariate. For index variants identified in the combined analysis of males and females, we tested for sex-differential effects using the following formula $Z_{diff}=\left(\hat{\beta_{1}}-\hat{\beta_{2}}\right)/\sqrt{se_{1}^{2}+se_{2}^{2}}$, where β_1_ represents the female, β_2_ the male effect and se_1_ and se_2_ the respective standard errors.^55^ Significant sex differences were identified with a two-sided test after Bonferroni-correction on the number of tested variants (p_SIA_ < 0.05). For loci where a significant sex interaction was identified for the index variant and multiple independent variants were identified in the sex-combined analysis, we repeated this analysis with conditioned statistics.

### Body mass index interaction analysis

We investigated variant-BMI interactions for genome-wide significant index variants in their respective best-associated phenotype. For this purpose, the genetic model was extended with an interaction term between gene-dosage and log-transformed mean-centred BMI. Single study GWAS with the extended model was performed for KORA, LIFE-Heart, LIFE-Adult, and the Sorbs study, as described in the section ‘Single-study genome-wide association analysis’, using Plink 2.0.^38^ Fixed-effect meta-analysis of the interaction effect was performed with the function ‘rma’ from the package ‘metafor’ (v. 4.8-0).^42^

### Mendelian randomisation mediation analysis

Mendelian randomisation (MR) mediation analysis was applied to estimate the causal effect of lanosterol on CAD mediated by cholesterol (indirect effect) and the causal direct effect.^56^ As instrument for lanosterol we selected rs12916, as the corresponding candidate gene *HMGCR* is directly involved in the synthesis of lanosterol, reducing the risk of horizontal pleiotropy. As instruments for cholesterol, we used 46 variants published by Surakka et al., excluding variants without genome-wide significance or cytobands overlapping with lanosterol loci identified by our study (downloaded on November 27th, 2025).^57^ Summary statistics for total cholesterol were taken from the same publication. Summary statistics for CAD were obtained from Aragam et al.^54^

First, the effect of lanosterol on total cholesterol (a) was estimated with the Wald ratio, using the first two terms of the delta method expansion to calculate variance.^58^ Secondly, the effect of total cholesterol on CAD (b) was estimated with inverse variance-weighted MR as implemented in the R-package ‘MendelianRandomization’ (v. 0.10.0).^59^^,^^60^ Thirdly, the Wald ratio estimate was used to calculate the total effect (c) of lanosterol on CAD. Then, the indirect effect is the product of a and b, while the direct effect is the difference between total and indirect effect ($c^{\prime }=c-a\times b$). Significance of the indirect effect was determined with a two-sided Sobel test (α = 0.05), comparing the test statistic to the normal distribution.^61^^,^^62^ Significance of the direct effect was tested with a two-sided Z-test (α = 0.05).

### Candidate gene lookup

We performed a lookup of candidate genes of sterol esterification, namely *LCAT*, *ACAT*, *SOAT1*, and *SOAT2* by searching for suggestive associations (p < 10^−6^) of SNPs in a ±500 kb window around these genes. Gene positions in hg19 coordinates were taken from GeneCards (v. 5.19).^51^^,^^52^

### Explained variance

We calculated the variance explained by all genome-wide significantly associated independent variants using the formula $r^{2}=\beta^{2}/\left(\beta^{2}+N\times \text{se}{\left(\beta \right)}^{2}\right)$, where β is the estimated effect of a variant in the meta-analysis, se(β) the standard error of the effect estimate, and N the total sample size.^63^ Conditional statistics were used if multiple independent variants were present at a locus. In case independent variants changed across phenotypes at a locus but conditioned signals were colocalized, the independent variants from the best-associated phenotype were considered as index variants for the other phenotypes. The sum of explained variances of independent variants associated with a specific trait resulted in the total genetically explained variance.

### Power analysis

We performed power analyses for free lanosterol and desmosterol to free lanosterol ratios to investigate the impact of differing sample sizes. Power calculation was performed with the function ‘genpwr.calc’ from the R-package ‘genpwr’ (v. 1.0.4),^64^ assuming an additive genetic model. Standard deviations of phenotypes were calculated in LIFE-Heart, the largest study where all phenotypes were present.

### Figure creation

Genome-wide significant associations were visualised in a circos-diagram with the R package ‘circlize’ (v. 0.4.15).^65^ Regional association (RA)-plots of genome-wide significantly associated loci were created with a custom script using European populations of 1000 Genomes phase 1 version 3^34^ as LD reference and the genetic map created from all populations in 1000 Genomes phase 1 version 3 as reference for recombination frequencies. Gene positions were taken from Ensembl.^47^ Forest-plots were created with the R-package ‘forestplot’ (v. 3.1.3).^66^ Heatmaps included in the supplement are created with the ‘pheatmap’ package (1.0.12).^67^ Other figures were created with ggplot2 (v. 3.5.2).^68^

### Ethics

All KORA participants have given written informed consent. The study was approved by the Ethics Committee of the Bavarian Medical Association and complies with the Declaration of Helsinki. Written informed consent was obtained from all participants of the LIFE-Heart study before taking part in the study. The study meets the ethical standards of the Declaration of Helsinki and has been approved by the University of Leipzig's ethics committee (Reg.-No. 276/05-ek) and is registered at ClinicalTrials.gov (NCT number NCT00497887). All participants of the LIFE-Adult-Study gave written informed consent to participate in the study. The procedures were conducted according to the Declaration of Helsinki and approved by the University of Leipzig's ethics committee (Reg.-No. 263-2009-14122009). The LURIC study was approved by the institutional review board of the ethics committee of the Landesärztekammer Rheinland-Pfalz (Reg.-No. 1997-203) and was performed in adherence to the principles of the Declaration of Helsinki. All subjects gave written informed consent. Informed consent has been given by all participants before taking part in the Sorbs study. The Sorbs study was approved by the ethics committee of the University of Leipzig (Reg.-No. 088-2005) and is in accordance with the Declaration of Helsinki. Individuals participating in YFS gave written informed consent. The YFS study complies with the Declaration of Helsinki and was approved by the local ethics committee of the Hospital District of Southwest Finland and the regional Ethics Committee of the Expert Responsibility area of Tampere University Hospital, Finland.

We used the STREGA checklist when writing our manuscript.^69^

### Role of funders

The funding sources had no role in the study design, data collection, data analyses, interpretation, or manuscript preparation.

## Results

### Genome-wide association meta-analysis

We performed a GWAMA of 14 traits related to three stages of cholesterol biosynthesis, namely lanosterol, desmosterol, and cholesterol. The combination of six studies resulted in a total sample size of up to 10,519 individuals. A detailed overview of trait-wise case numbers is shown in Supplementary Table S3. After QC, between 8,481,851 (free desmosterol to free cholesterol ratio) and 8,994,509 (total cholesterol) variants remained (see Supplementary Table S4). Neither genomic inflation factors, ranging from 0.98 (free desmosterol to free cholesterol ratio) to 1.03 (free lanosterol), nor quantile-quantile-plots indicated inflation of test statistics (see Supplementary Figure S2 and Supplementary Table S4). A sensitivity analysis, excluding individuals treated with lipid-lowering medication, showed high correlation with effect estimates based on inclusion of treatment status as a binary covariate (see Supplementary Figure S3).

Significant heritability was observed for five traits (see Supplementary Table S5), namely free lanosterol (h^2^ = 0.15, p = 0.005), free cholesterol (h^2^ = 0.14, p = 0.001), total cholesterol (h^2^ = 0.13, p = 0.001), esterified cholesterol (h^2^ = 0.11, p = 0.004), and free lanosterol to total cholesterol ratio (h^2^ = 0.11, p = 0.031). We tested these traits for pairwise genetic correlation (see Supplementary Table S6). Cholesterol traits (esterified, total, and free cholesterol) were highly correlated with each other on the genetic level (r_g_ > 0.9, max(p) = 8.06 × 10^−78^). Genetic correlations between free lanosterol and cholesterol traits were weaker, ranging from 0.53 (free lanosterol and free cholesterol, p = 0.007) to 0.77 (free lanosterol and esterified cholesterol, p = 2.06 × 10^−4^). A strong genetic correlation was observed between free lanosterol and free lanosterol to total cholesterol ratio (r_g_ = 0.97, p = 1.83 × 10^−178^). No significant genetic correlation was observed between lanosterol to total cholesterol ratio and the cholesterol traits (esterified, total, and free cholesterol, respective p-values are 0.09, 0.11, and 0.35).

### Locus definition

**Fig. 1:**
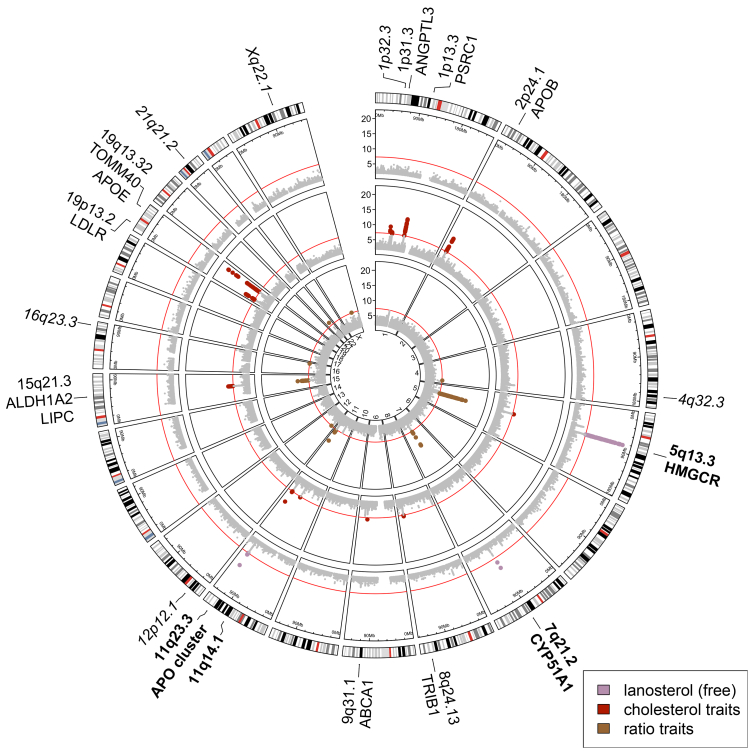
Circos-plot of zoosterol traits and their genetic associations. Circles correspond to different groups of investigated phenotypes: outer circle—free lanosterol, middle circle—cholesterol traits (free, esterified, and total cholesterol), inner circle—zoosterol ratios (free to esterified cholesterol, free desmosterol to free lanosterol, total desmosterol to free lanosterol, free desmosterol to free cholesterol, free lanosterol to total cholesterol). The x-axis represents the chromosomal position of variants. The respective y-axes show the negative log-transformed two-sided p-values (Z-test) for the test of association. Only variants with negative log-transformed p-values > 2 are depicted. Negative log-transformed p-values > 20 were set to 20. Red lines represent the genome-wide significance threshold (p < 5 × 10^−8^). Genome-wide significantly associated variants are highlighted through trait-specific colouring. The outer ring shows the UCSC hg19 cytobands with centromeres marked red and the nucleolus organising regions marked blue. Cytobands and candidate genes of genome-wide significant loci are annotated on the outside. Loci without support for the index-variant by neighbouring variants are italicised and not considered further. Loci with associations not previously reported in the GWAS catalogue are shown in bold.

With our locus definition procedure, we identified 18 genome-wide significantly associated loci. An overview of these associations is shown in Fig. 1 and Supplementary Table S7. RA-plots of these loci are shown in Supplementary Figure S4. Of these 18 loci, six showed a solitary index variant without support by neighbouring variants. To reduce the risk of false positive associations, these loci were discarded, although the plausible gene locus 1p32.3 (associated with free, esterified, and total cholesterol), including the *PCSK9* gene, was one of them.

An overview of the twelve supported loci is shown in Table 1. Effect directions were consistent across studies, eight loci showing identical directions across all studies and four loci including only a single study with opposite effect direction (see Supplementary Figure S5). A comparison of results from fixed effect and random effects model for index variants with I^2^ > 0.4 showed loss of genome-wide significance for four loci, namely 1p13.3, 8q24.13, 15q21.3, 19p13.2 (see Supplementary Table S8). A meta-regression, including the sterol measurement procedure as binary moderator, did not reveal significant effects after FDR correction (minimal q-value = 0.388, see Supplementary Table S9). To assess the influence of lipid-lowering medication, a subset of four studies was meta-analysed, including lipid-treatment as a covariate in the regression model in setting 1 and excluding treated individuals in setting 2. No significant effect differences were observed (see Supplementary Table S10), although four loci (1p31.3, 8q24.13, 9q31.1, 19p13.2) lost genome-wide significance in both settings. No significant genetic interactions with BMI were observed for the index variants (see Supplementary Table S11).

**Table 1:** Independent variants with genome-wide significant association to zoosterol traits.

Region	Cytoband	Index variant	Pos	EA	AA	Best association	P-value	Beta	SE	N	EAF	Info	I^2^	Gene
1	1p31.3	rs11208009	63,195,692	T	C	cht	1.55e-08	0.013	0.002	10,519	0.61	0.99	0.25	*ANGPTL3*
2	1p13.3	rs7528419	109,817,192	G	A	cht	1.44e-13	−0.021	0.003	10,519	0.22	0.95	0.52	*PSRC1*
3	2p24.1	rs11279109	21,266,774	G	GGCAGCGCCA	chf	6.27e-13	0.022	0.003	10,087	0.30	0.97	0	*APOB*
**4**	**5q13.3**	rs12916	74,656,539	C	T	laf	4.22e-21	0.063	0.007	8786	0.42	0.99	0.42	*HMGCR*
**5**	**7q21.2**	rs190126802	91,162,879	C	T	laf/cht	8.69e-15	0.236	0.030	7024	0.01	0.79	0.26	
**5**	**7q21.2**	rs2299239	91,654,617	T	C	laf/cht	4.38e-08	0.035	0.006	8786	0.37	0.97	0	*CYP51A1*
6	8q24.13	rs2954032	126,493,392	G	A	chf	2.09e-08	−0.017	0.003	10,087	0.71	0.99	0.54	*TRIB1*
7	9q31.1	rs4149307	107,589,744	T	C	chf	1.38e-08	0.021	0.004	10,087	0.15	0.97	0.14	*ABCA1*
**8**	**11q14.1**	rs17629703	79,700,923	G	C	chf	2.81e-08	−0.042	0.008	10,087	0.04	0.76	0.38	
**9**	**11q23.3**	rs964184	116,648,917	C	G	laf	6.18e-13	−0.067	0.009	8786	0.86	0.99	0.26	APO cluster
10	15q21.3	rs2043082	58,674,308	A	G	chf/che	1.34e-12	0.010	0.001	10,087	0.33	0.99	0.52	*ALDH1A2*
10	15q21.3	rs588136	58,730,498	T	C	chf/che	3.24e-11	−0.010	0.002	10,087	0.79	0.97	0.54	*LIPC*
11	19p13.2	rs6511720	11,202,306	T	G	cht	1.88e-11	−0.026	0.004	10,519	0.10	0.91	0.67	*LDLR*
12	19q13.32	rs59007384	45,396,665	T	G	cht	2.44e-08	0.017	0.003	10,519	0.21	0.75	0	*TOMM40*
12	19q13.32	rs7412	45,412,079	T	C	che	6.64e-30	−0.052	0.005	10,087	0.09	0.75	0	*APOE*

Variants are sorted according to their genetic position. Novel associations are highlighted in bold. An underscore indicates the presence of multiple independent variants at a locus, resulting in additional lines for this locus. For independent variants, conditional statistics are presented. The best-associated phenotype was chosen based on the presented statistics. Candidate genes are proposed based on secondary analyses as mentioned in the text. Phenotype abbreviations: laf, free lanosterol; def, free desmosterol; dee, esterified desmosterol; det, total desmosterol; chf, free cholesterol; che, esterified cholesterol; cht, total cholesterol. Column abbreviations: Pos, genetic position (hg19); EA, effect allele; AA, alternative allele; SE, standard error; N, sample size; EAF, effect allele frequency; Info, imputation info score.

**Fig. 2:**
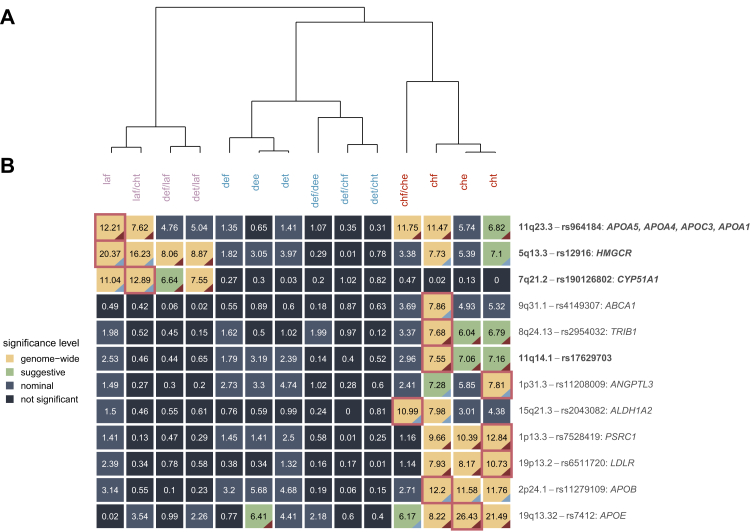
Comparison of association signals across phenotypes for genome-wide significant loci. A: Hierarchical clustering of investigated zoosterol traits based on partial regression coefficients. Colours indicate results from cluster assignment. B: Negative log-transformed two-sided p-values (Z-test) of index-variants across investigated zoosterol traits for genome-wide significant loci. Colours indicate significance level (genome-wide: p < 5 × 10^−8^, suggestive: p < 1 × 10^−6^, nominal: p < 0.05). A red border marks best-associated traits for each locus. The colour of the triangle indicates the direction of effects with at least suggestive significance, red: negative effect, blue: positive effect. For each locus the cytoband, index variant, and candidate genes for the index variant are presented. Associations not previously reported in the GWAS catalogue with regard to the best-associated trait are marked in bold. Loci are ordered according to similar pattern of phenotype association. Phenotype abbreviations: laf, free lanosterol; def, free desmosterol; dee, esterified desmosterol; det, total desmosterol; chf, free cholesterol; che, esterified cholesterol; cht, total cholesterol.

Associations were found for eight traits, namely free lanosterol, free, esterified, and total cholesterol, free to esterified cholesterol ratio, free lanosterol to total cholesterol ratio, free desmosterol to free lanosterol ratio, and total desmosterol to free lanosterol ratio. Fig. 2 shows a comparison of the association strength of index variants across hierarchically clustered traits. Clustering of phenotypes revealed three clusters, corresponding to stages of cholesterol biosynthesis. The first cluster (see Fig. 2A, violet traits) involved lanosterol-related traits (free lanosterol, free lanosterol to total cholesterol, free desmosterol to free lanosterol, and total desmosterol to free lanosterol). The second cluster (see Fig. 2A, blue traits) involved traits connected to desmosterol (free, esterified, and total desmosterol, free to esterified desmosterol, free desmosterol to free cholesterol, total desmosterol to total cholesterol), while the third cluster (see Fig. 2A, red traits) included traits connected to cholesterol (free, esterified, and total cholesterol, free to esterified cholesterol ratio). The index variants of three loci (5q13.3, 7q21.2, and 11q23.3) showed the strongest association with traits belonging to the lanosterol cluster and were consequently associated with other traits within this cluster. The index variants of loci 5q13.3 and 11q23.3 were further associated with traits in the cholesterol cluster, being the only variants for which a cross–cluster association was observed. The index variants of nine loci (1p31.3, 1p13.3, 2p24.1, 8q24.13, 9q31.1, 11q14.1, 15q21.3, 19p13.2, and 19q13.32) showed their strongest association with traits belonging to the cholesterol cluster. At all of these loci, with the exception of 9q31.1, we observed at least suggestive association with other traits of the cholesterol cluster. No genome-wide significant associations with traits belonging to the desmosterol cluster were found. To verify identity of signals for loci associated with multiple phenotypes, we performed colocalisation analyses for each locus associated with more than one trait. Colocalisation was supported in all cases (see Supplementary Figure S6).

### Single locus results

For each of the twelve loci, we conducted COJO analysis to identify further independent associations (see Supplementary Tables S12 and S13). We assigned candidate genes based on bioinformatic annotation of 99% CSs (see Supplementary Table S14) and annotated variants within the CSs with the CADD score to evaluate functional relevance,^48^ LD with eQTLs, and traits previously reported for these variants (see Supplementary Table S15). We further tested loci for colocalisation with eQTLs (see Supplementary Table S16) and CAD (see Supplementary Table S17), investigated the presence of sex-differential effects for index variants (see Supplementary Table S18), and calculated the variance explained by each independent variant (see Supplementary Table S19). This section describes results for the individual loci, starting with four loci that showed novel associations with respect to the best-associated trait.

#### Novel associations

Four novel associations were detected. Three of these (5q13.3, 7q21.2, 11q23.3) were primarily associated with lanosterol related traits, while the fourth one (11q14.1) was associated with free cholesterol. At locus 5q13.3 the index variant rs12916 was most strongly associated with free lanosterol (p = 4.22 × 10^−21^, explained variance: 1.00%) and was further associated with free desmosterol to free lanosterol ratio (explained variance: 0.80%), free lanosterol to total cholesterol ratio (explained variance: 0.79%), total desmosterol to free lanosterol ratio (explained variance: 0.62%), and free cholesterol (explained variance: 0.31%, see Fig. 2B and Supplementary Table S19). Previously reported associations of this variant included several phytosterol to lanosterol ratios like free brassicasterol to free lanosterol ratio,^70^ as well as multiple cholesterol related phenotypes, including but not limited to total cholesterol, LDL-C,^71^ and free cholesterol.^72^ The index variant is a modifier of the 3′-UTR of the gene *HMGCR*. The CS for the free lanosterol association included 15 variants, including one variant with high CADD-value (rs10038095, CADD = 16.2), an intronic variant of *HMGCR*. Colocalisation between the observed free lanosterol association and an eQTL for *HMGCR* was supported in skeletal muscle (PP(H4) = 0.93). Of note, colocalisation with CAD risk was supported for this locus (PP(H4) = 1.00).

The index variant at locus 7q21.2 was rs190126802. Conditional analysis supported rs2299239 as an additional independent variant. Both variants are primarily associated with the free lanosterol to total cholesterol ratio (rs190126802: p_cond_ = 8.69 × 10^−15,^ explained variance: 0.90%, rs2299239: p_cond_ = 4.38 × 10^−8^, explained variance: 0.35%). Rs190126802 was further associated with total desmosterol to free lanosterol ratio (explained variance: 0.74%) and free lanosterol (explained variance: 0.66%) but for these phenotypes no independent variants were found. A previous study reported an association of rs6465351, a variant in LD to rs2299239 (r^2^ = 0.66, based on LDlink in European populations),^73^ with HDL-cholesterol.^74^ Here, we did not observe associations with cholesterol level traits. The observed absence of these traits is further verified by the lack of colocalisation between our conditional signals for lanosterol to total cholesterol ratio and lipid traits measured by the Global Lipids Genetics Consortium (maximal PP(H4) = 0.48, observed between signal for rs2299239 and triglycerides).^3^ An RA-plot based on the conditional statistics is shown in Supplementary Figure S7. As the secondary independent variant rs2299239 was located close to the locus boundary, we repeated COJO analysis after extending this locus by 500 kb downstream. Rs2299239 remained the strongest associated variant after conditioning on rs190126802 (see Supplementary Figure S8). The CSs for rs190126802 and rs2299239 were large, including 283 and 177 variants, respectively. Only a small fraction of included variants had a reasonably high probability of being causal, the CS for rs190126802 including 27 variants with PP > 1% and the CS for rs2299239 including 25 such variants. The index variant rs190126802 is an intronic variant of the non-coding RNA RP11-142A5.1 (ENSG00000235450). Three variants with PP > 1% in the CS of rs190126802 had CADD > 10, all are located in RP11-142A5.1. Neither colocalisation with CAD (PP(H4) = 0.03) nor eQTLs was supported. The index variant rs190126802 showed initially a significant sex interaction that did not withstand multiple testing correction (adjusted p_SIA_ = 0.40, see Supplementary Table S18). The two nearest genes to the secondary variant rs2299239 were *AKAP9* and *CYP51A1*. Colocalisation between the association of rs2299239 with free lanosterol to total cholesterol ratio and an eQTL of *CYP51A1* and in skeletal muscle (PP(H4) = 0.89) as well as an eQTL for *AKAP9* in whole blood (PP(H4 = 0.89) was supported. As for the index variant rs190126802, the secondary variant rs2299239 was also not previously reported for CAD or sterol related traits and no colocalisation with CAD (PP(H4) = 0.00) was observed. The CS included three high CADD variants with PP > 1%, all located within or near *AKAP9*.

The index variant at 11q23.3, rs964184, was primarily associated with free lanosterol (p = 6.18 × 10^−13^, explained variance: 0.59%) and showed additional genome-wide significant associations with free to esterified cholesterol ratio (explained variance: 0.49%), free cholesterol (explained variance: 0.48%), and free lanosterol to total cholesterol ratio (explained variance: 0.35%). The 99% CS was comprised only of the index variant. The index variant is located in the 3′-UTR of *ZNF259*, also known as *ZPR1*. Nearby is a cluster of four genes encoding the apolipoproteins APOA1, APOC3, APOA4, and APOA5. *APOA5* was the gene closest to the index variant with a distance of 11 kb. The index variant was previously reported for several lipid traits, including total cholesterol,^71^ and CAD.^75^ Consequently, we observed colocalisation with CAD (PP(H4) = 1.00). Colocalisation with an eQTL for *ZNF259* was observed (PP(H4) = 0.94) in whole blood and no support was found for colocalisation with eQTLs of APO-genes.

Out of the four loci with novel associations, locus 11q14.1 was the only one not primarily associated with lanosterol related traits. The index variant rs17629703 was associated with free cholesterol only (p = 2.81 × 10^−8^, explained variance: 0.30%). 2831 variants were included in the 99% CS, but only seven had PP > 1% and therefore a sufficiently high probability of being causal. These seven variants are located in a gene desert, devoid of protein-coding genes. Only RP11-885L14.1 (ENSG00000254471), a processed pseudogene, was found nearby. No support was found for colocalisation with CAD (PP(H4) = 0.01) or eQTLs.

#### Known loci

We replicated eight loci that were previously described for various cholesterol traits. Three loci were primarily associated with total cholesterol (1p31.3, 1p13.3, 19p13.2), three loci primarily associated with free cholesterol (2p24.1, 8q24.13, 9q31.1), one locus primarily associated with free to esterified cholesterol ratio (15q21.3), and one primarily associated with esterified cholesterol (19q13.32). When searching these loci for additional independent variants, such variants were found at 15q21.3 and 19q13.32. RA-plots for these two-loci based on the conditional statistics are shown in Supplementary Figure S7. We also tested for colocalisation with CAD and searched for sex-differential effects. Additional insights are summarised in the following.

For 15q21.3 we found the index variant rs2043082 and the secondary variant rs588136, both with the strongest association to free to esterified cholesterol ratio (rs2043082: p_cond_ = 1.34 × 10^−12^, explained variance: 0.49%, rs588136: p_cond_ = 3.24 × 10^−11^, explained variance: 0.44%). Further, both variants were genome-wide significantly associated with free cholesterol, explaining 0.35% (rs2043082) and 0.30% (rs588136) of variance, respectively. Rs2043082 is an intronic variant of *ALDH1A2*. Rs588136 is an intronic variant of two genes, *ALDH1A2* and *LIPC*. For this variant we also observed a colocalisation with a *LIPC* eQTL in liver tissue (PP(H4) = 0.97).

The two independent variants at 19q13.32 were rs7412 and rs59007384. The strongest association for rs7412 was observed with esterified cholesterol (p_cond_ = 6.64 × 10^−30^, explained variance: 1.50%) and further genome-wide significant associations were observed for total and free cholesterol (respective explained variances: 1.20% and 0.33%). Notably, this variant also showed suggestive association with esterified desmosterol (p = 3.89 × 10^−7^), the only signal we observed for desmosterol related traits. Rs7412 is a missense mutation of *APOE*, and together with rs429358 it determines functionally different isoforms.^76^ We observed a sex-differential effect for this variant (adjusted p_SIA_ = 1.52 × 10^−3^, see Supplementary Table S18), supporting previously found sex-differential effects of *APOE* variants.^77^ The sex-differential effect was also significant when conditioning on the other independent variant rs59007384 (p_SIA_ = 2.75 × 10^−4^, see Supplementary Table S20). This other independent variant, rs59007384, showed its strongest association with total cholesterol (p_cond_ = 2.44 × 10^−8^, explained variance: 0.34%) and was also associated with esterified cholesterol (explained variance: 0.36%). Rs59007384 is an intronic variant of the *TOMM40* gene. Colocalisation with eQTLs of *APOC1* (PP(H4) = 0.96) in the adrenal gland and *APOC1P1* in liver (PP(H4) = 0.83) were observed. When using conditional statistics, no significant sex interaction was observed (p_SIA_ = 0.92, see Supplementary Table S20). Conditioning on rs7412 and rs429358, the variants determining the *APOE* haplotype, lead to a loss of significance for the association of rs59007384 (total cholesterol: p_cond_ = 0.04). It can therefore not be excluded that isoforms of *APOE* might cause the secondary association.

Colocalisation with CAD was observed for five of the cholesterol loci, 1p13.3 (PP(H4) = 0.99), 8q24.13 (PP(H4) = 0.82), 15q21.3 with rs588136 (PP(H4) = 1.00), 19p13.2 (PP(H4) = 0.95), and 19q13.32 for both independent variants (rs7412: PP(H4) = 1.00, rs59007384: PP(H4) = 0.99).

### Mendelian randomisation mediation analysis

MR mediation analysis was used to investigate the mediation of lanosterol's effect on CAD by total cholesterol. The SNP rs12916, located near *HMGCR*, was used as instrument for lanosterol and 46 previously published variants were selected as instruments for total cholesterol (see Supplementary Table S21). The results are shown in Supplementary Table S22. Significant positive total effects of lanosterol (c = 0.45, SE(c) = 0.09, p = 1.13 × 10^−6^) and total cholesterol on CAD were observed (b = 0.35, SE(b) = 0.04, p = 3.01 × 10^−15^), as expected. The indirect effect, i.e. the effect of lanosterol on CAD mediated by total cholesterol, was 0.46 (SE(ab) = 0.08) and differed significantly from 0 (p = 3.67 × 10^−8^). In contrast, the direct effect of lanosterol on CAD was not significant (c’ = −0.01, SE(c’) = 0.13, p = 0.939).

### Total explained variances

An overview of the total variance of zoosterol traits explained by independent variants with genome-wide significant associations is given in Supplementary Table S23. Explained variance was highest for cholesterol related traits, namely free cholesterol (4.22%), total cholesterol (3.26%), and esterified cholesterol (3.10%), followed by the lanosterol-related traits free lanosterol to total cholesterol (2.39%) and free lanosterol (2.24%). The explained variance was lowest for three ratio traits, namely free to esterified cholesterol (1.42%), total desmosterol to free lanosterol (1.36%), and free desmosterol to free lanosterol (0.80%).

### Candidate gene lookup

We searched for associated variants near *LCAT*, *ACAT*, *SOAT1*, and *SOAT2*, which are involved in sterol esterification. An overview of all investigated variants is shown in Supplementary Table S24. No nearby variants showed at least suggestive significance (p < 10^−6^).

## Discussion

**Fig. 3:**
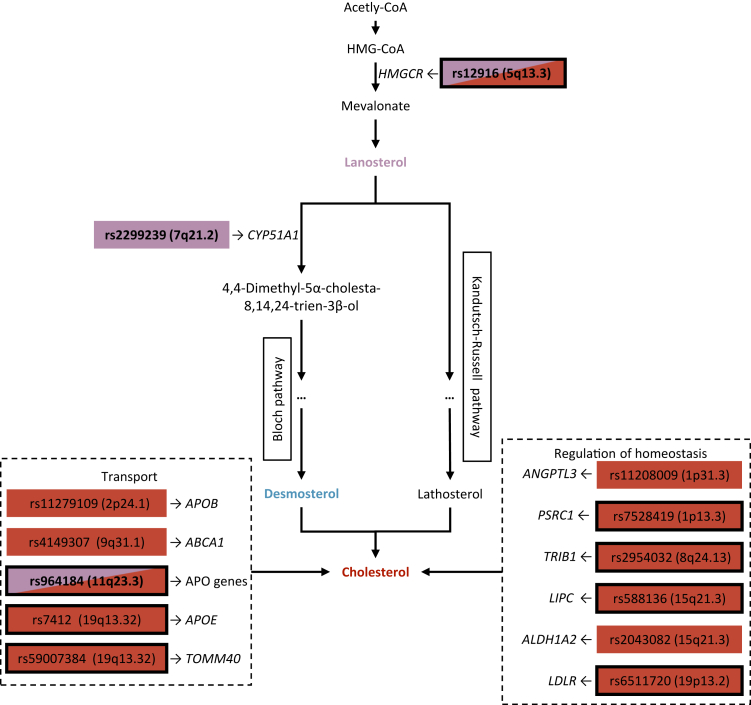
Functional impact of variants associated with zoosterol traits with regard to cholesterol biosynthesis, transport, and regulation of homoeostasis. Shown are genome-wide significantly associated variants (p < 5 × 10^−8^, Z-test) and respective candidate genes, categorised according to their biological function. Investigated zoosterols are highlighted through colouring. The colour of associated variants indicates associated traits: red—cholesterol related traits, violet—lanosterol related traits. Associations not previously reported in the GWAS catalogue are highlighted in bold. A black border around variants indicates colocalisation with coronary artery disease.

We performed a GWAMA of 14 zoosterol-related traits representing different stages of cholesterol synthesis, consisting of seven zoosterol level traits and seven ratio traits. We identified twelve genome-wide significantly associated loci, including four novel associations. Three of the respective loci were primarily associated with lanosterol-related traits and nearby genes had direct functional relation to cholesterol biosynthesis (see Fig. 3). In contrast, the fourth locus was associated with free cholesterol. The eight previously reported associations all involved cholesterol-related traits and did not show genome-wide significant associations with traits related to lanosterol or desmosterol. Colocalisation with CAD was supported for seven loci, including the two novel associations for free lanosterol at 5q13.3 and 11q23.3, indicating clinical relevance. These two novel lanosterol associations were observed at loci previously associated with cholesterol. For both loci, explained variance for free lanosterol was higher than for cholesterol related traits, suggesting that previously observed associations are indeed driven by changes in lanosterol concentration. Therefore, our results allow for a more precise functional interpretation of observed associations.

The locus 5q13.3 was genome-wide significantly associated with all four lanosterol-related traits. Additionally, we observed a genome-wide significant association with free cholesterol. As candidate gene we propose *HMGCR*. *HMGCR* encodes 3-hydroxy-3-methyl-glutaryl-coenzyme A (HMG-CoA) reductase, which converts HMG-CoA to mevalonic acid, the precursor to lanosterol. This reaction is also known to be the rate-limiting step of the cholesterol synthesis.^78^ The observed associations are consistent with this choice of candidate gene, as the reaction step is located upstream of lanosterol in the synthesis pathway and should therefore affect lanosterol and cholesterol traits equally, as is supported by the association with lanosterol and cholesterol related traits. The explained variance for free lanosterol was higher than that for free cholesterol, supporting the notion that the association is driven by lanosterol. Noteworthy, no associations with desmosterol concentration were observed. This can be explained by the Kandutsch-Russell pathway as an alternative to the Bloch pathway, allowing for cholesterol synthesis without desmosterol as an intermediate. Both pathways are activated in a tissue-specific manner and in liver contribute comparably to cholesterol synthesis.^12^ Previous studies of *HMGCR* hypothesised that the index variant exhibits its effect on cholesterol concentration through changes of *HMGCR* expression levels.^79^^,^^80^ We observed colocalisation with an *HMGCR* eQTL in skeletal muscle, but not in any of the other investigated tissues. In muscle tissue, cholesterol primarily originates from local synthesis and excess cholesterol can be exported into blood.^8^^,^^81^ The lack of colocalisation with an eQTL in liver indicates regulatory differences between liver and muscle.^82^ In a previous investigation of phytosterols, we reported associations for several phytosterol to lanosterol ratios at this locus, the strongest association being observed for free brassicasterol to free lanosterol ratio.^70^ Due to the lack of associations with phytosterol concentrations, we assumed these signals to be caused by changes in lanosterol concentration. The association with lanosterol concentration in the present study further supports this assumption.

The index variant of 11q23.3 was located in the 3′-UTR of *ZNF259*, also known as *ZPR1*, a zinc finger protein involved in cell cycle progression and with no reported functional connection to cholesterol biosynthesis or transport. In contrast, the nearby gene *APOA5* encodes APOA5, which is not only a constituent of lipoproteins, relevant for lipid transport, but can also regulate lipid concentrations by indirectly activating lipoprotein lipase mediated degradation of triglyceride containing particles.^83^ The 99% CS of 11q23.3 included variants near additional APO genes. We therefore propose the APO cluster as candidates. Lipoproteins, especially HDL, are shown to not only transport cholesterol but also sterol precursors like desmosterol.^84^ Consequently, we observed associations with free lanosterol, free to esterified cholesterol, free cholesterol, and free lanosterol to total cholesterol. Explained variance for free lanosterol was higher than the explained variance for free cholesterol, indicating that changes in lanosterol concentrations drive the associations with cholesterol-related traits. It seems contradictory that associations with lanosterol concentration in absence of associations with desmosterol concentration do not result in significant associations with desmosterol to lanosterol ratios, as has been observed for loci 5q13.3 and 7q21.2. This could be a result of power differences between traits, as free desmosterol to free lanosterol showed lower power than free lanosterol due to the reduced sample size, although total desmosterol to free lanosterol ratio showed similar power to free lanosterol (see Supplementary Figure S9). As the index variant rs964184 was neither part of a coding region nor colocalized with eQTLs of APO-genes, the mechanism of its regulatory effect remains unclear. The association might be caused by an untagged variant near the APO-genes linked to our index variant.

The locus 7q21.2 was only associated with lanosterol-related traits. The ratio between free lanosterol to total cholesterol was the best-associated phenotype, indicating a change in reaction equilibrium. Since the index variant was not associated with cholesterol traits, the ratio's association is primarily driven by lanosterol. We suppose that the mechanism behind this association compensates for a reduced concentration or activity of an enzyme downstream of lanosterol in the synthesis pathway. For the independent variant rs2299239, there were two potential candidate genes, *AKAP9* and *CYP51A1*. *AKAP9* encodes a scaffolding protein which binds the regulatory subunit of protein kinase A. There is no obvious connection to lipid traits. As candidate gene for rs2299239, we therefore propose *CYP51A1*. This gene encodes sterol 14alpha-demethylase, an enzyme needed for the removal of the 14α-methyl group from lanosterol during cholesterol biosynthesis.^85^ The selected candidate gene aligns well with the observed associations, because it is responsible for the reaction directly downstream of lanosterol. Further, lanosterol as well as its demethylation intermediate 3β-hydroxy-lanost-8-en-32-al are known regulators of HMGCR,^86^^,^^87^ making compensatory mechanisms possible. Additionally, members of the cytochrome P450 family, to which *CYP51A1* belongs, are known to be post transcriptionally regulated by lncRNAs.^88^ A lncRNA was found in the CS of the index variant of this locus, rs190126802, although evidence for an interaction with *CYP51A1*'s transcript is lacking. Due to the lack of protein-coding genes with relation to cholesterol biosynthesis near variants of the CS of rs190126802, we were not able to select a candidate gene for this variant. This locus was the only one of the lanosterol-associated loci, where no colocalisation with CAD was observed. This is likely caused by the lack of associations with cholesterol traits, supporting the assumption that lanosterol itself does not affect CAD.

11q14.1 was the only locus with a novel association where the strongest association was observed for a cholesterol-related rather than a lanosterol-related trait. We acknowledge the lack of a protein-coding gene at this locus, as well as the fact that cholesterol is a heavily investigated trait, where novel associations found in smaller studies need to be carefully considered. The strength of this association for free cholesterol was barely below the genome-wide significance threshold (p = 2.81 × 10^−8^) and the correlated traits total and esterified cholesterol only reached suggestive significance (p = 6.92 × 10^−8^, respectively 8.70 × 10^−8^). Therefore, this might be a borderline signal, remaining below the significance threshold in earlier studies. Nevertheless, this locus should be considered tentative and requires validation by future studies.

For the two known loci 15q21.3 and 19q13.32 we identified independent variants and therefore performed candidate gene assignment per independent variant. The index variant rs2043082 of locus 15q21.3 was located within *ALDH1A2*. The encoded enzyme is responsible for the rate-limiting step during synthesis of retinoic acid,^89^ a regulator of cholesterol homoeostasis.^90^ We therefore selected *ALDH1A2* as candidate gene. The secondary variant rs588136 was additionally an intronic variant of *LIPC*. This gene encodes lipase C, which catalyses the hydrolysis of triglycerides and regulates cholesterol homoeostasis.^91^ Due to the observed colocalisation with an eQTL of *LIPC* in liver, we selected *LIPC* as candidate gene for rs588136. The index variant of 19q13.32, rs7412, is a missense mutation of *APOE*. Experiments in mice have shown that *APOE* knock-out increases plasma desmosterol levels, potentially to enable hippocampal cholesterol synthesis as compensation for the reduced cholesterol transport.^92^ Consequentially, we selected *APOE* as candidate gene. The second independent variant at 19q13.32, rs59007384, is an intronic variant of *TOMM40*, coding for a subunit of a translocase localised in the outer mitochondrial membrane. We also observed colocalisations with the apolipoprotein coding gene *APOC1*. We selected *TOMM40* as a candidate gene due the localisation of the variant.

For the remaining cholesterol-associated loci, our candidate gene assignment overlapped with previously proposed genes (see Table 1, Supplementary Table S15). We selected *ANGPTL3* (1p31.3), involved in regulation of lipid metabolism,^93^, ^94^, ^95^ *PSRC1* (1p13.3), regulating mitotic spindle dynamics and potentially modulating cholesterol transport,^96^ *APOB* (2p24.1),^71^ an apolipoprotein, *TRIB1* (8q24.13), involved in pathway signalling and shown to affect lipid concentrations in knock-out experiments,^97^ *ABCA1* (9q31.1), regulating cholesterol efflux,^98^ and *LDLR* (19p13.2), coding for the low-density lipoprotein receptor.

Colocalisation with CAD was observed for several loci, including two associated with lanosterol, suggesting clinical relevance of these loci. We therefore performed an MR mediation analysis to explore the causal relation between lanosterol, cholesterol, and CAD. Lanosterol showed a significant total effect on CAD, but when separating into a direct and an indirect effect mediated trough total cholesterol, only the indirect effect was significant. Therefore, the effect of lanosterol on CAD is likely fully mediated by total cholesterol and lanosterol does not constitute an independent risk factor.

In this study, the investigation of cholesterol precursors enabled us to perform functional mapping of cholesterol associated loci. Further, the identification of a suitable genetic instrument for lanosterol allowed us to estimate direct and indirect effects on CAD with an MR mediation analysis. Still, certain limitations need to be addressed. First, due to the restricted availability of mass-spectrometric platforms for high-throughput measurement of non-cholesterol sterols, our sample size is limited especially when compared to large consortia investigating cholesterol and its lipoprotein-bound fractions. This also limited our insights into the role of sex. Sample size of sex-stratified GWAS is approximately halved and was therefore deemed too small to be used for reliable detection of sex-specific variants, which might not have been detected in the combined analysis. Second, our insights into cholesterol biosynthesis are limited by the small range of zoosterols measured, with desmosterol being the only intermediate of the Bloch pathway. The inclusion of precursors of lanosterol from the mevalonate pathway and additional intermediates of cholesterol biosynthesis, especially from the Kandutsch-Russell pathway, is therefore of high future interest but analytically challenging. Third, sterol concentrations were measured in blood serum, allowing for a minimal invasive extraction. Although blood is the most relevant biosample to investigate the effects of cholesterol on CAD, sterol concentrations in blood might not be representative for the tissues involved in cholesterol synthesis, especially when intermediates accumulate in the cell and are not exported. Additionally, the rates by which intermediate sterols are exported from cells into the blood might vary from compound to compound.^84^ Fourth, we included lipid-lowering medication as a binary covariate in the association analysis, therefore not considering individual dosage schemes or mechanistic differences between specific medications. Fifth, our meta-analysis combined studies using two different methods for zoosterol measurement. Although no significant moderator effects were observed, several loci lost genome-wide significance when using random effects rather than fixed effect meta-analysis model. While respective loci represent replications of previously known cholesterol loci, i.e. they can be assumed to be true positives, a potential bias of the differing measurement platforms cannot be fully ruled out. Sixth, two-sample MR was used to estimate the causal effect of free lanosterol on total cholesterol in the mediation analysis. While this approach reduces bias compared to one-sample MR, bias can arise from sample overlap between the used studies. As the sample overlap between the two studies is below 3.3% and bias in a two-sample MR is proportional to the overlap, we believe this effect to be negligible.^99^

In conclusion, we performed a GWAMA of sterol traits, representing different stages of cholesterol biosynthesis. Three novel associations for lanosterol and one for cholesterol were identified. With our study, we demonstrated that the investigation of intermediate sterol species could improve our understanding of the genetic regulators of sterol biosynthesis. In particular, stronger and biologically more interpretable associations were discovered. Also our results show that further investigations of lanosterol are promising, as we estimated a chip-based heritability for free lanosterol of 15%, while our identified variants currently explain 2.24% of this variance.

## Contributors

Franz Förster: formal analysis, interpretation of results, writing–original draft; Katrin Horn: single study quality control, implementation of analyses, writing–critical review; Janne Pott: single study quality control, implementation of analyses, writing–critical review; Graciela E. Delgado: single study analysis, writing–critical review; Marcus E. Kleber: single study analysis, writing–critical review; Winfried März: management of contributing studies, writing–critical review; Angela P. Moissl-Blanke: writing–critical review; Günther Silbernagel: zoosterol measurements, writing–critical review; Melanie Waldenberger: management of contributing studies, writing–critical review; Harald Grallert: management of contributing studies, writing–critical review; Annette Peters: management of contributing studies, writing–critical review; Christian Gieger: management of contributing studies, writing–critical review; Ronny Baber: writing–critical review; Holger Kirsten: implementation of analyses, writing–critical review; Markus Loeffler: management of contributing studies writing–critical review; Berend Isermann: writing–critical review; Joachim Thiery: management of contributing studies, writing–critical review; Peter Kovacs: genotyping, writing–critical review; Anke Tönjes: management of contributing studies, writing–critical review; Michael Stumvoll: management of contributing studies, writing–critical review; Helena Gylling: management of contributing studies, writing–critical review; Mika Kähönen: management of contributing studies, writing–critical review; Terho Lehtimäki: management of contributing studies, writing–critical review; Pashupati Prasad Mishra: single study analysis, writing–critical review; Olli Raitakari: management of contributing studies, writing–critical review; Uta Ceglarek: study design, zoosterol measurements, supervision, writing–critical review; Markus Scholz: study design, supervision, management of contributing studies, writing–editing and critical review. All authors read and approved the final version of the manuscript. Franz Förster, Katrin Horn, Uta Ceglarek, and Markus Scholz had access to the data for verification.

## Data sharing statement

Individual level data is subject to data privacy restrictions and will not be shared publicly. Data access for individual level data can be requested directly from responsible data access committees of the participating studies. Summary statistics of the meta-analysis have been deposited in the Leipzig Health Atlas under accession code 8YGW0GPGPY-8 (https://www.health-atlas.de/lha/8YGW0GPGPY-8). Analysis scripts are provided on GitHub (https://github.com/GenStatLeipzig/GWAMA_zoosterol).

## Declaration of interests

Marcus E. Kleber is an employee of Synlab Holding Deutschland GmbH. Winfried März reports grants and personal fees from AMGEN, grants and personal fees from Sanofi, grants and personal fees from Amryt Pharmaceuticals, grants and personal fees from Abbott Diagnostics, grants and personal fees from Akzea Therapeutics, grants from Novartis Pharma GmbH, personal fees from SOBI, employment with SYNLAB Holding Deutschland GmbH, and board membership of DACH Society Prevention of Cardiovascular Disease, outside the submitted work. Günther Silbernagel reports grants and contracts from the Austrian Science Fund (FWF, KLI-1117) and Sanofi AG, minor stock or stock-options for Novartis, Novo Nordisk, Roche, Sanofi, and receipt of equipment, materials, drugs, medical writing, gifts or other services from Eli Lilly and Company. Uta Ceglarek received grants or contracts from Roche Diagnostics, the DFG, as well as the BMBF, and is a member of the DGKL board. Markus Scholz received funding from Owkin for a project not related to this research. The remaining authors declare no competing interests.
