# Supplementary material for: A genome-wide association meta-analysis of cholesterol synthesis intermediates identifies three associations for lanosterol
Source: eBioMedicine. 2026 Jan 30;124:106144. doi: 10.1016/j.ebiom.2026.106144 (PMC12876664; doi:10.1016/j.ebiom.2026.106144)
Supplement: Supplementary Figures [file mmc2.pdf]

Supplementary Figures

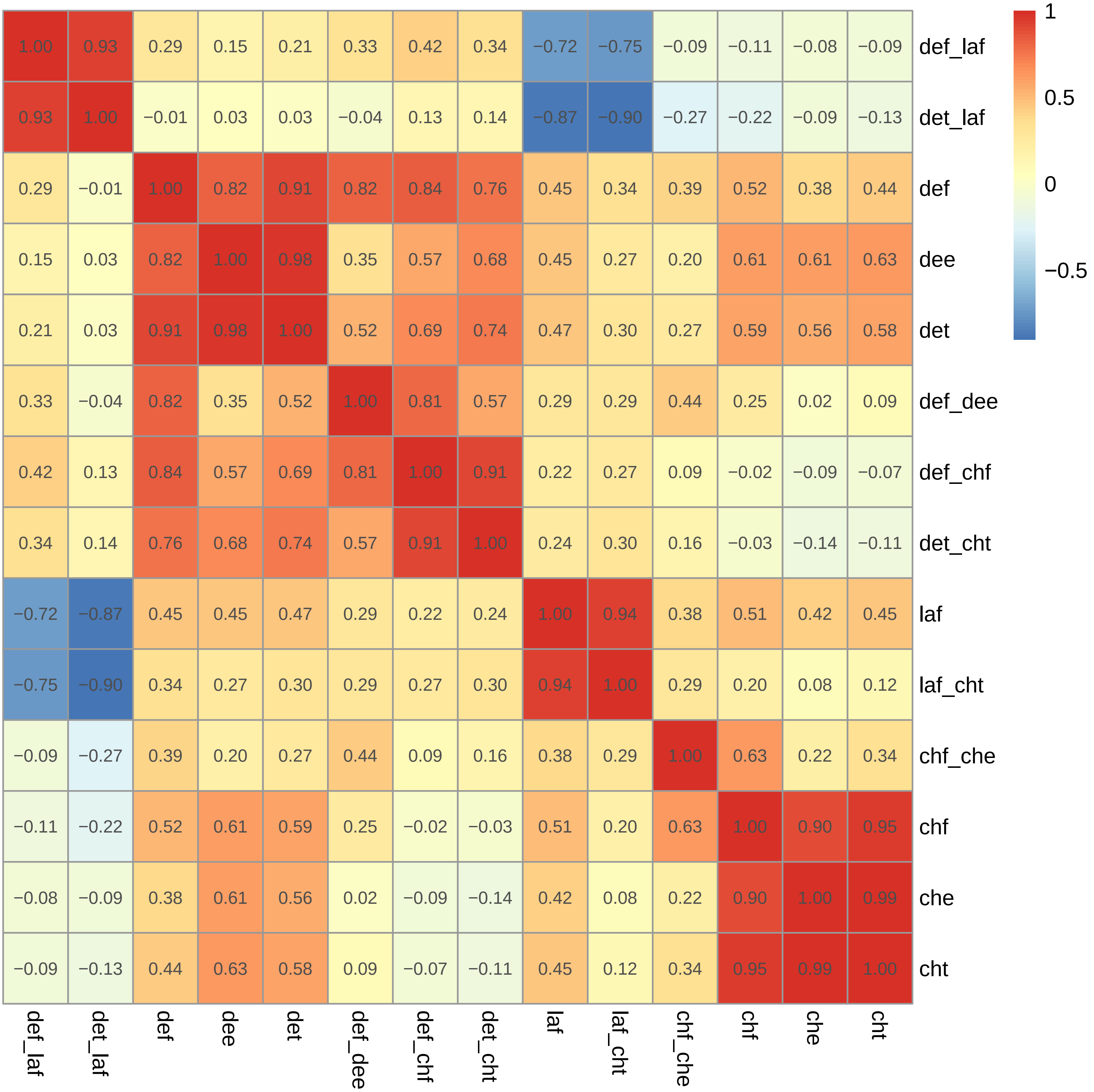

Sup. Fig. S1: Pearson correlation coefficients obtained from pairwise partial correlation of zoosterol traits. Phenotype abbreviations: laf - free lanosterol, def - free desmosterol, dee - esterified desmosterol, det - total desmosterol, chf - free cholesterol, che - esterified cholesterol, cht - total cholesterol. Ratio traits are indicated with an underscore.

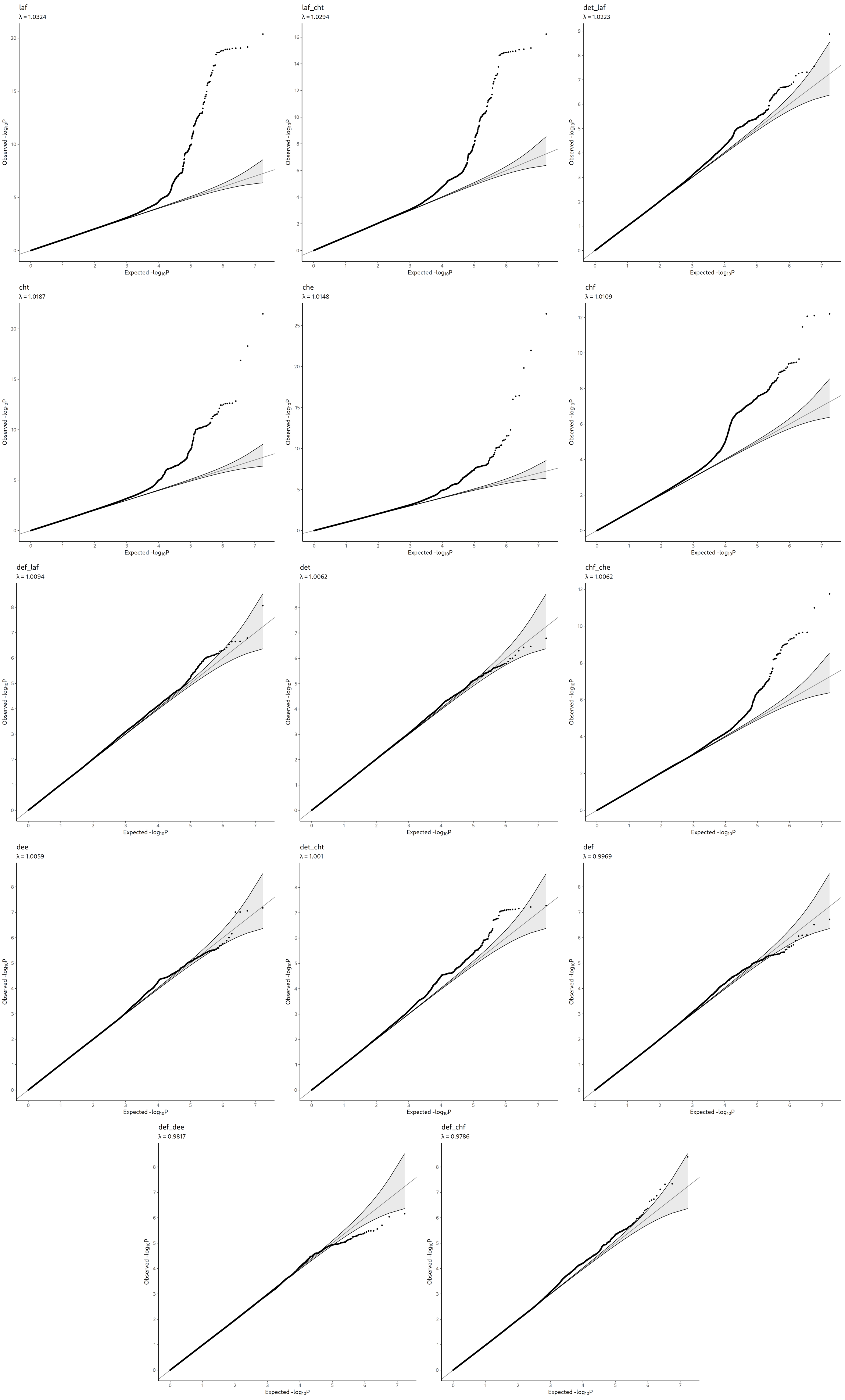

Sup. Fig. S2: Quantile-Quantile-plots and genomic-inflation factors λ for investigated traits. Only variants passing quality-control criteria were included in the calculation of λ. Traits are ordered from left to right in order of decreasing λ. Phenotype abbreviations: laf - free lanosterol, def - free desmosterol, dee - esterified desmosterol, det - total desmosterol, chf - free cholesterol, che - esterified cholesterol, cht - total cholesterol. Ratio traits are indicated with an underscore.

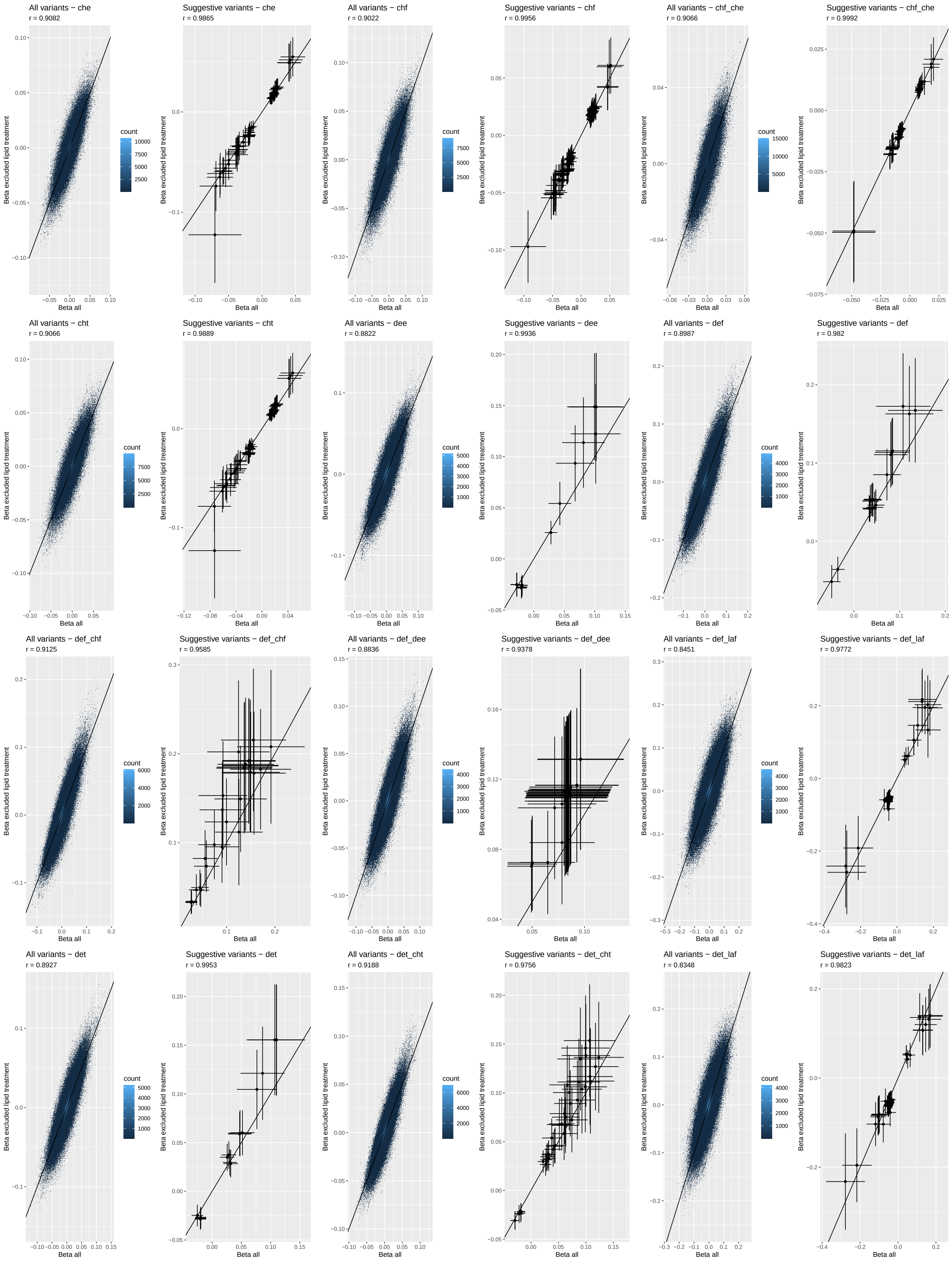
All variants − che
r = 0.9082
Suggestive variants − che
r = 0.9865
All variants − chf
r = 0.9022
Suggestive variants − chf
r = 0.9956
All variants − chf_che
r = 0.9066
Suggestive variants − chf_che
r = 0.9992
All variants − cht
r = 0.9066
Suggestive variants − cht
r = 0.9889
All variants − dee
r = 0.8822
Suggestive variants − dee
r = 0.9936
All variants − def
r = 0.8987
Suggestive variants − def
r = 0.982
All variants − def_chf
r = 0.9125
Suggestive variants − def_chf
r = 0.9585
All variants − def_dee
r = 0.8836
Suggestive variants − def_dee
r = 0.9378
All variants − def_laf
r = 0.8451
Suggestive variants − def_laf
r = 0.9772
All variants − det
r = 0.8927
Suggestive variants − det
r = 0.9953
All variants − det_cht
r = 0.9188
Suggestive variants − det_cht
r = 0.9756
All variants − det_laf
r = 0.8348
Suggestive variants − det_laf
r = 0.9823
Beta excluded lipid treatment
Beta all
count

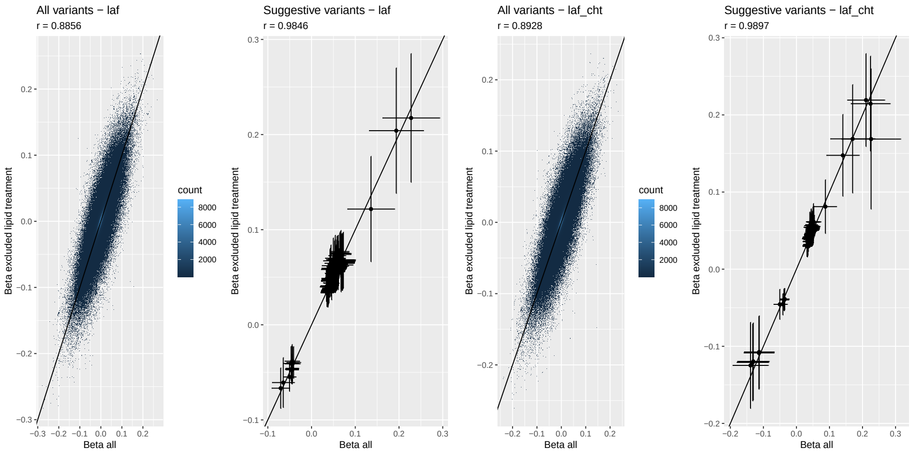

Sup. Fig. S3: Sensitivity analysis for the impact of lipid-lowering medication. Effect estimates of a meta-regression of all individuals including lipid-lowering medication as a covariate (Beta all) and a meta-regression excluding treated individuals (Beta excluded lipid treatment) are compared. Comparison and calculation of Pearson regression coefficients (r) was done for all quality-filtered variants and the subset of variants that reached suggestive significance ($p < 10^{-6}$) in either of the analysis settings. For suggestive variants, the 95% confidence interval is shown.

Region 1: rs11208009

trait: cht; Infoscore: 0.99; MAF: 0.39

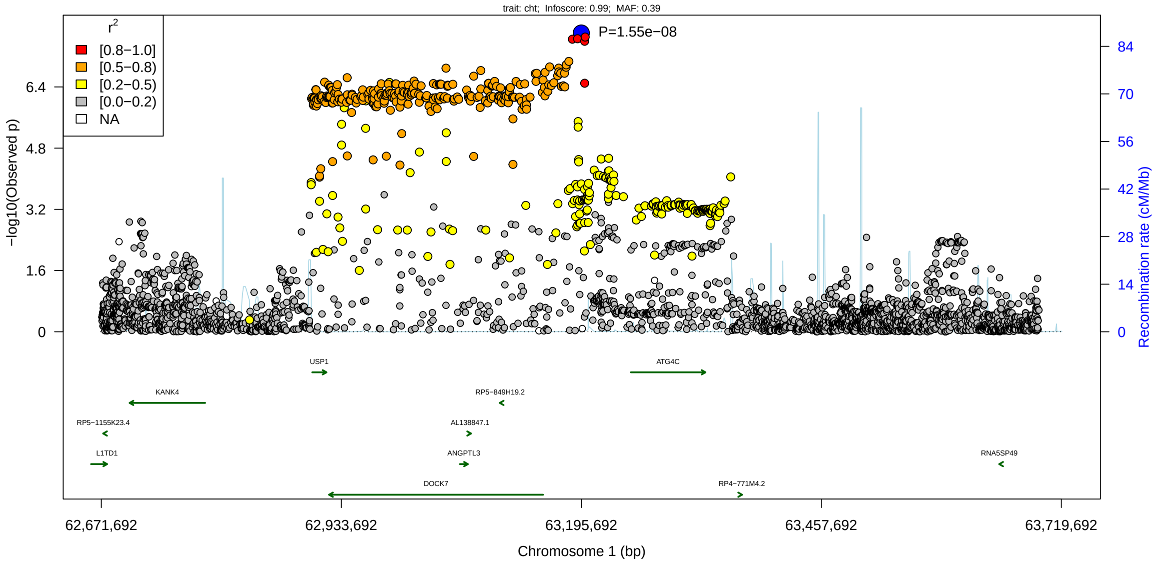

Region 2: rs7528419

trait: cht; Infoscore: 0.95; MAF: 0.22

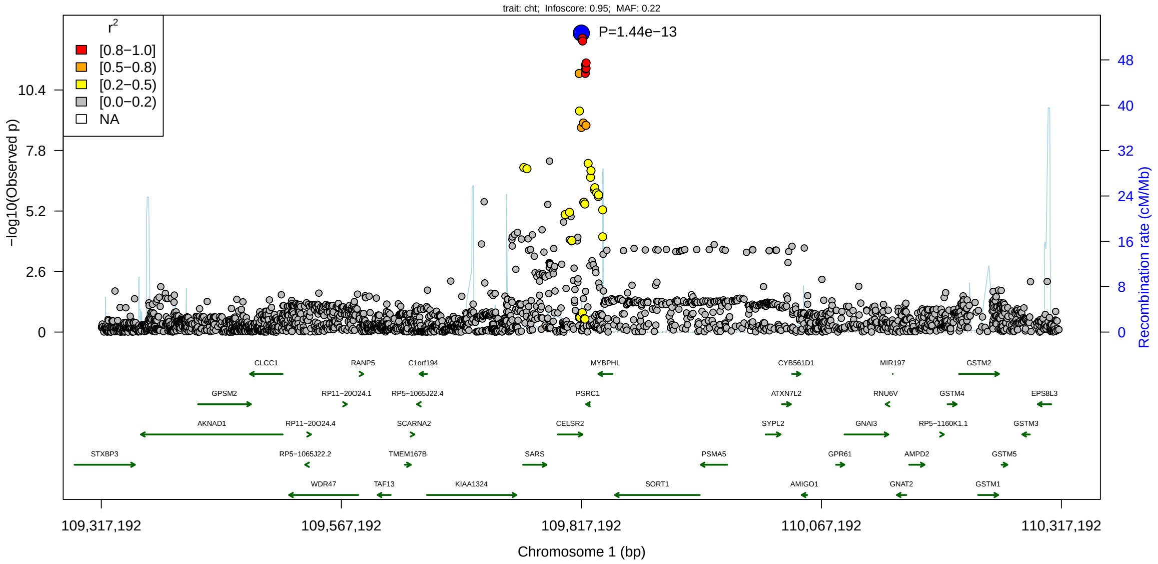

Region 3: chr2:21266774:D

trait: chf; Infoscore: 0.97; MAF: 0.3

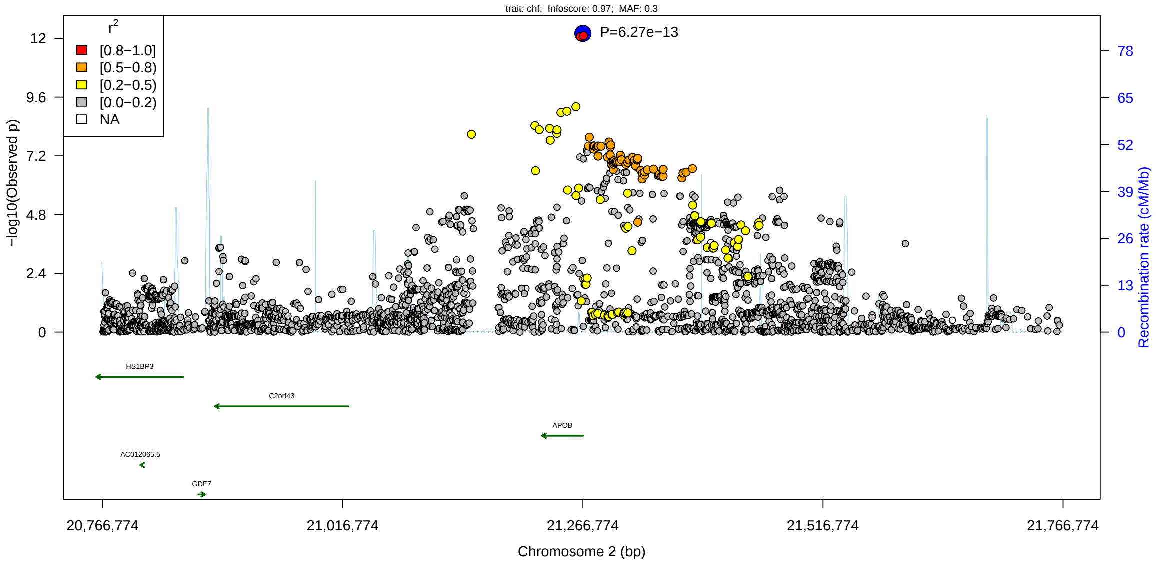

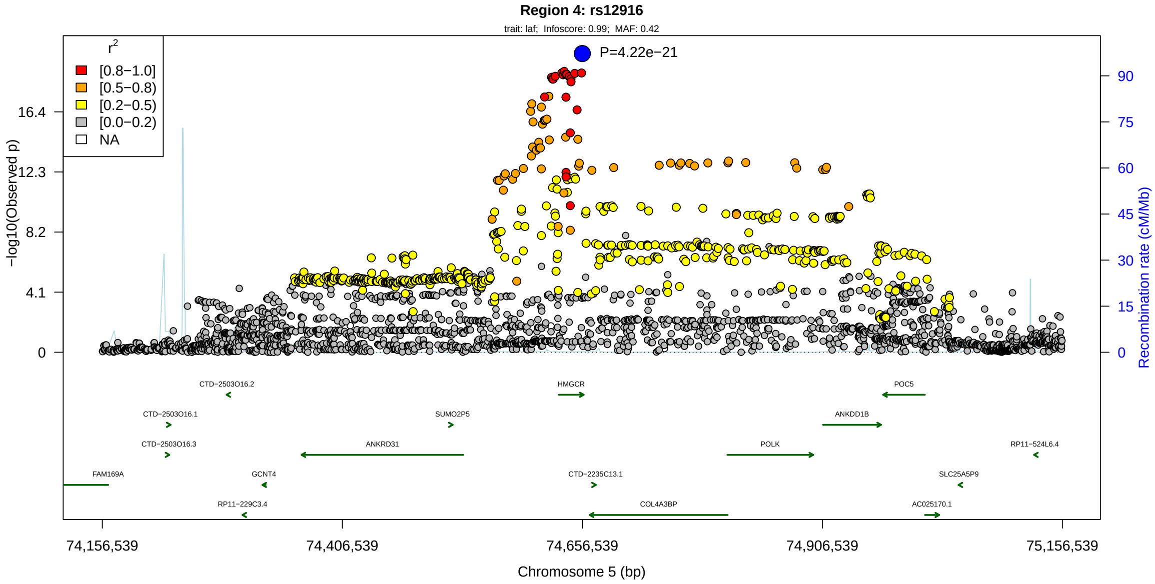
Region 4: rs12916
trait: laf; Infoscore: 0.99; MAF: 0.42
r2
[0.8−1.0]
[0.5−0.8]
[0.2−0.5]
[0.0−0.2]
NA
P=4.22e−21
−log10(Observed p)
16.4
12.3
8.2
4.1
0
Recombination rate (cM/Mb)
90
75
60
45
30
15
0
CTD−2503O16.2
HMGCR
POC5
CTD−2503O16.1
SUMO2P5
ANKDD1B
CTD−2503O16.3
ANKRD31
POLK
RP11−524L6.4
FAM169A
GCNT4
CTD−2235C13.1
SLC25A5P9
RP11−229C3.4
COL4A3BP
AC025170.1
74,156,539
74,406,539
74,656,539
74,906,539
75,156,539
Chromosome 5 (bp)

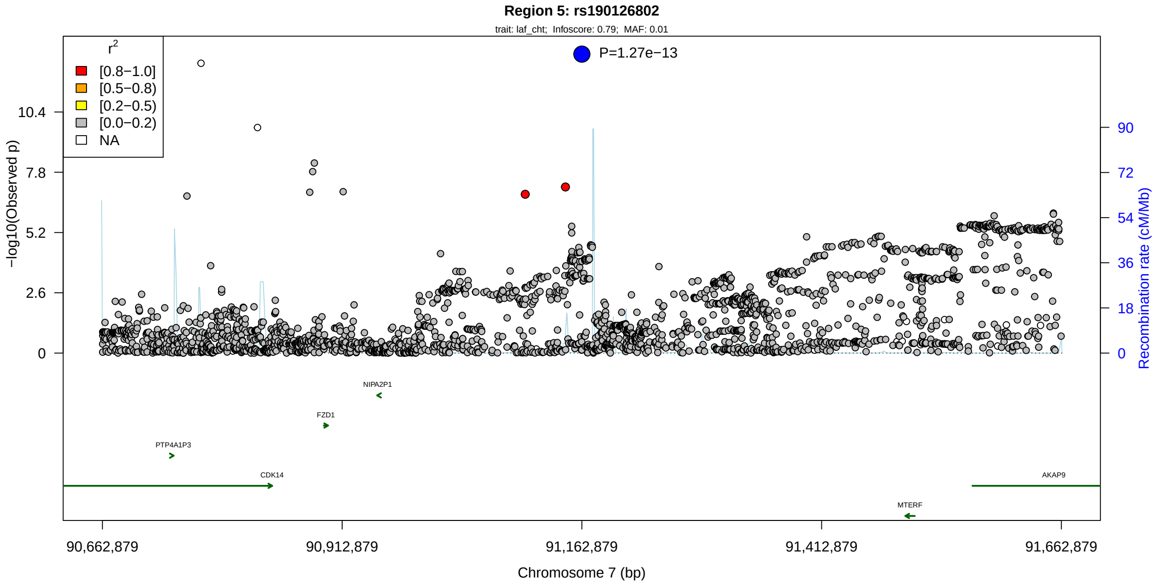
Region 5: rs190126802
trait: laf_cht; Infoscore: 0.79; MAF: 0.01
r2
[0.8−1.0]
[0.5−0.8]
[0.2−0.5]
[0.0−0.2]
NA
P=1.27e−13
−log10(Observed p)
10.4
7.8
5.2
2.6
0
Recombination rate (cM/Mb)
90
72
54
36
18
0
NIPA2P1
FZD1
PTP4A1P3
CDK14
AKAP9
MTERF
90,662,879
90,912,879
91,162,879
91,412,879
91,662,879
Chromosome 7 (bp)

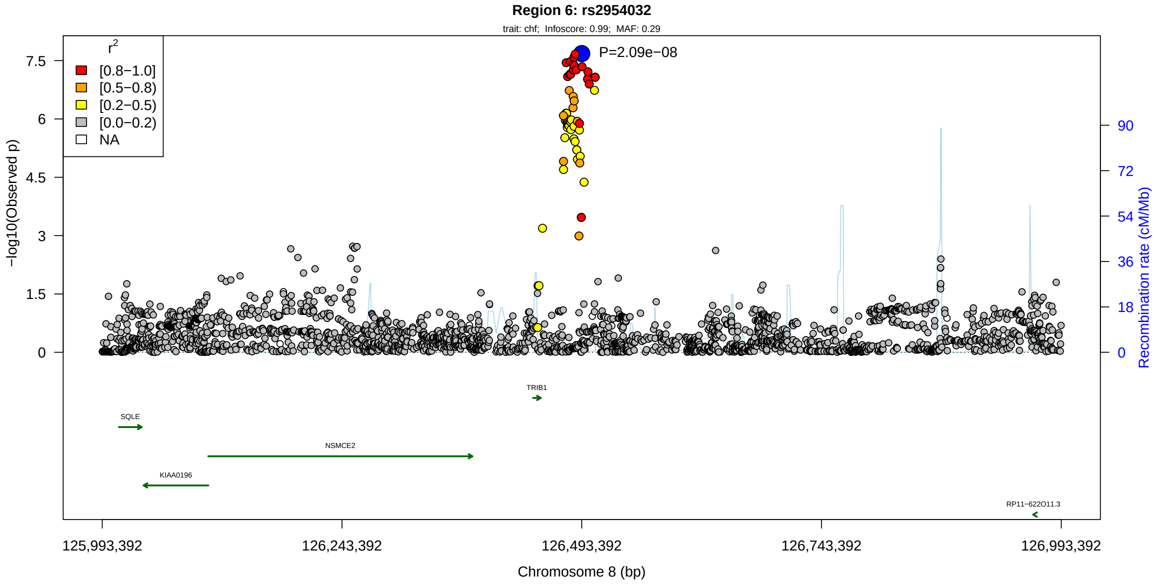
Region 6: rs2954032
trait: chf; Infoscore: 0.99; MAF: 0.29
r2
[0.8−1.0]
[0.5−0.8]
[0.2−0.5]
[0.0−0.2]
NA
P=2.09e−08
−log10(Observed p)
7.5
6
4.5
3
1.5
0
Recombination rate (cM/Mb)
90
72
54
36
18
0
TRIB1
SQLE
NSMCE2
KIAA0196
RP11−622O11.3
125,993,392
126,243,392
126,493,392
126,743,392
126,993,392
Chromosome 8 (bp)

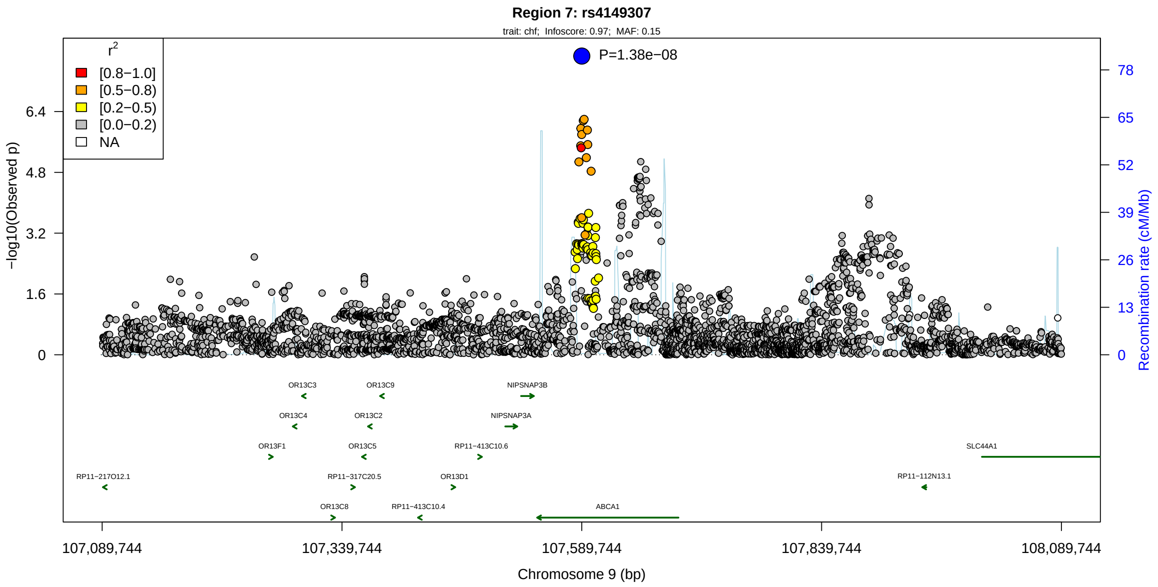
Region 7: rs4149307
trait: chf; Infoscore: 0.97; MAF: 0.15
P=1.38e−08
r2
[0.8−1.0]
[0.5−0.8)
[0.2−0.5)
[0.0−0.2)
NA
−log10(Observed p)
0
1.6
3.2
4.8
6.4
Recombination rate (cM/Mb)
0
13
26
39
52
65
78
OR13C3
OR13C9
NIPSNAP3B
OR13C4
OR13C2
NIPSNAP3A
OR13F1
OR13C5
RP11−413C10.6
SLC44A1
RP11−217O12.1
RP11−317C20.5
OR13D1
RP11−112N13.1
OR13C8
RP11−413C10.4
ABCA1
107,089,744
107,339,744
107,589,744
107,839,744
108,089,744
Chromosome 9 (bp)

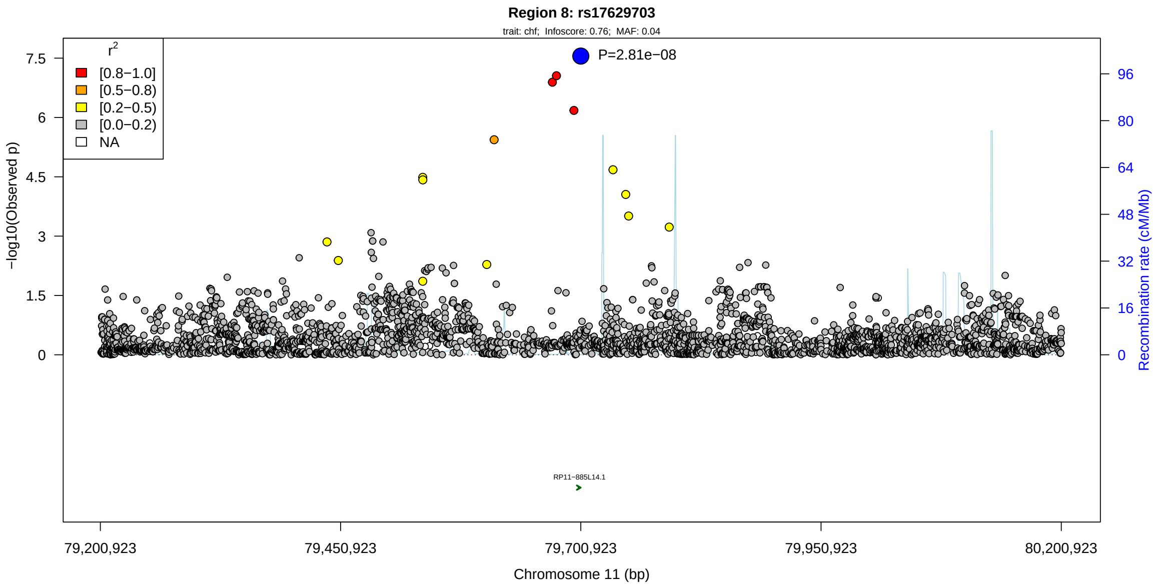
Region 8: rs17629703
trait: chf; Infoscore: 0.76; MAF: 0.04
P=2.81e−08
r2
[0.8−1.0]
[0.5−0.8)
[0.2−0.5)
[0.0−0.2)
NA
−log10(Observed p)
0
1.5
3
4.5
6
7.5
Recombination rate (cM/Mb)
0
16
32
48
64
80
96
RP11−885L14.1
79,200,923
79,450,923
79,700,923
79,950,923
80,200,923
Chromosome 11 (bp)

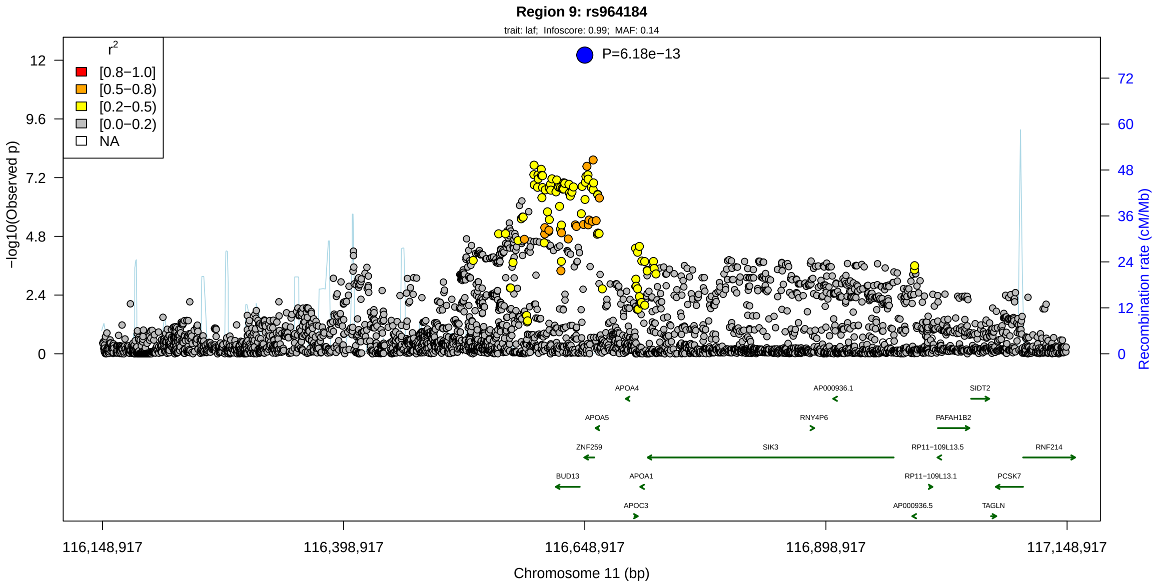
Region 9: rs964184
trait: laf; Infoscore: 0.99; MAF: 0.14
P=6.18e−13
r2
[0.8−1.0]
[0.5−0.8)
[0.2−0.5)
[0.0−0.2)
NA
−log10(Observed p)
0
2.4
4.8
7.2
9.6
12
Recombination rate (cM/Mb)
0
12
24
36
48
60
72
APOA4
AP000936.1
SIDT2
APOA5
RNY4P6
PAFAH1B2
ZNF259
SIK3
RP11−109L13.5
RNF214
BUD13
APOA1
RP11−109L13.1
PCSK7
APOC3
AP000936.5
TAGLN
116,148,917
116,398,917
116,648,917
116,898,917
117,148,917
Chromosome 11 (bp)

Region 10: rs2043082

trait: chf_che; Infoscore: 0.99; MAF: 0.33

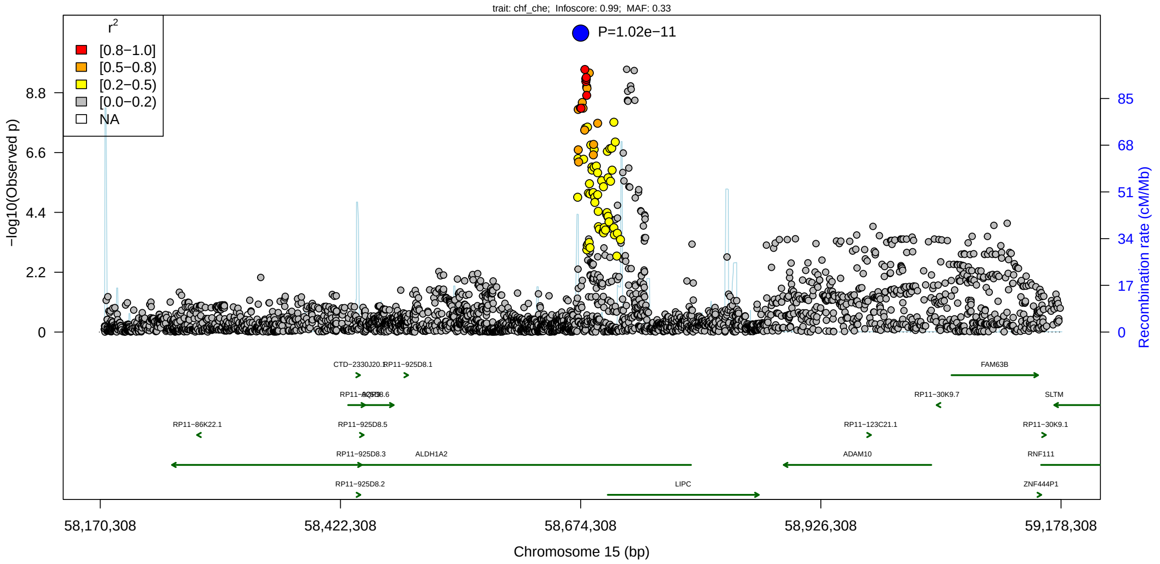

Region 11: rs6511720

trait: cht; Infoscore: 0.91; MAF: 0.1

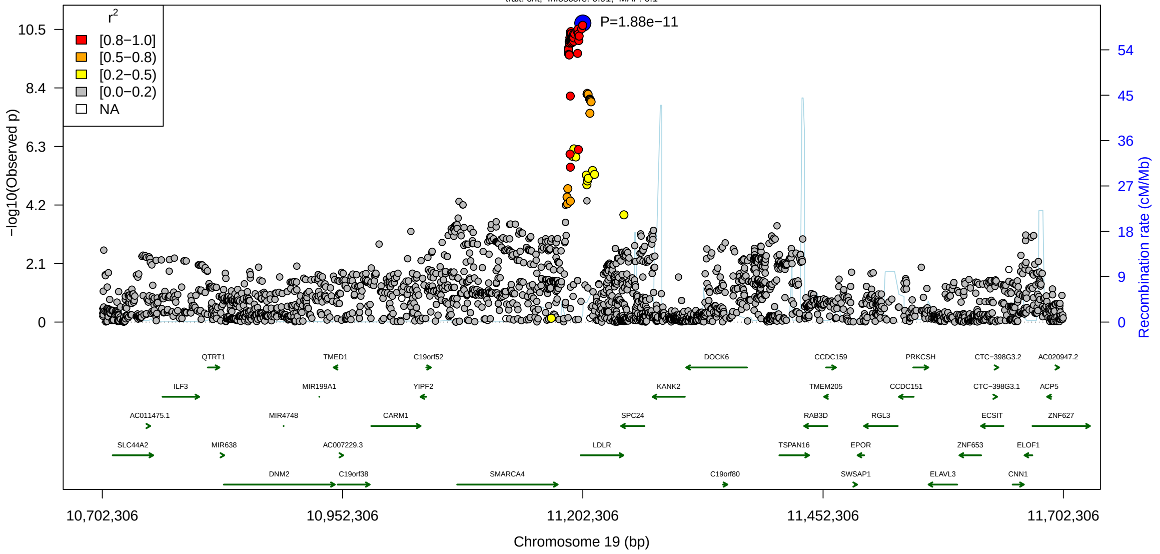

Region 12: rs7412

trait: che; Infoscore: 0.75; MAF: 0.09

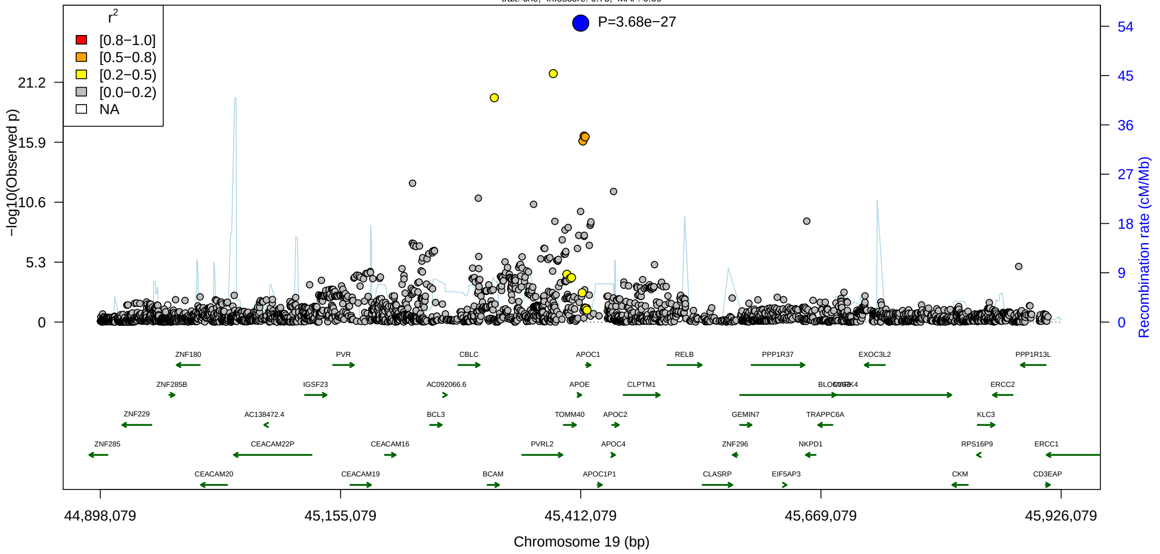

Unsupported Region U1: rs11591147

trait: cht; Infoscore: 0.87; MAF: 0.02

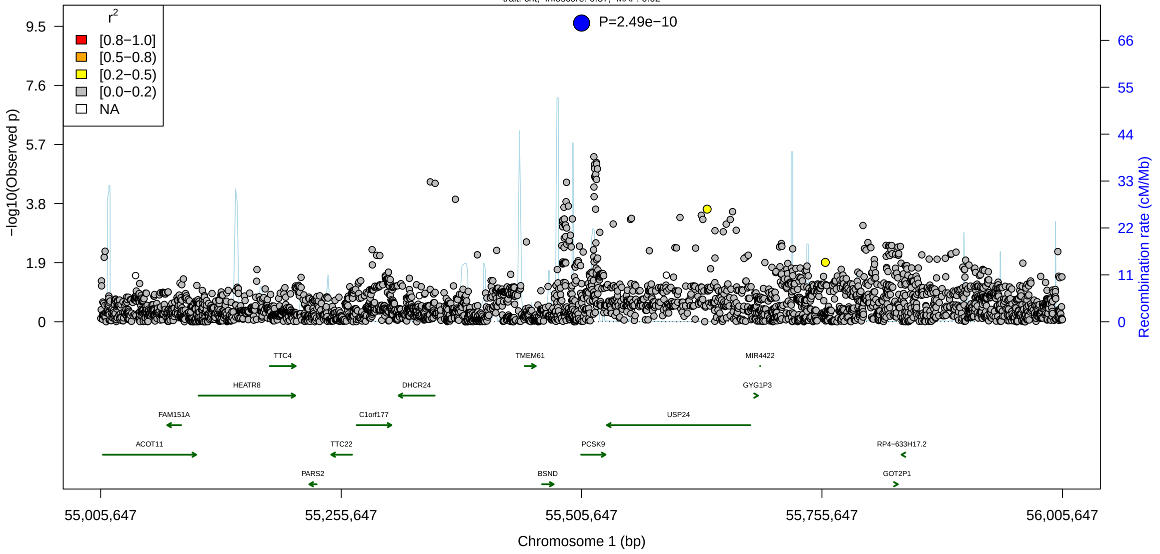

Unsupported Region U2: rs116343057

trait: chf_che; Infoscore: 0.73; MAF: 0.01

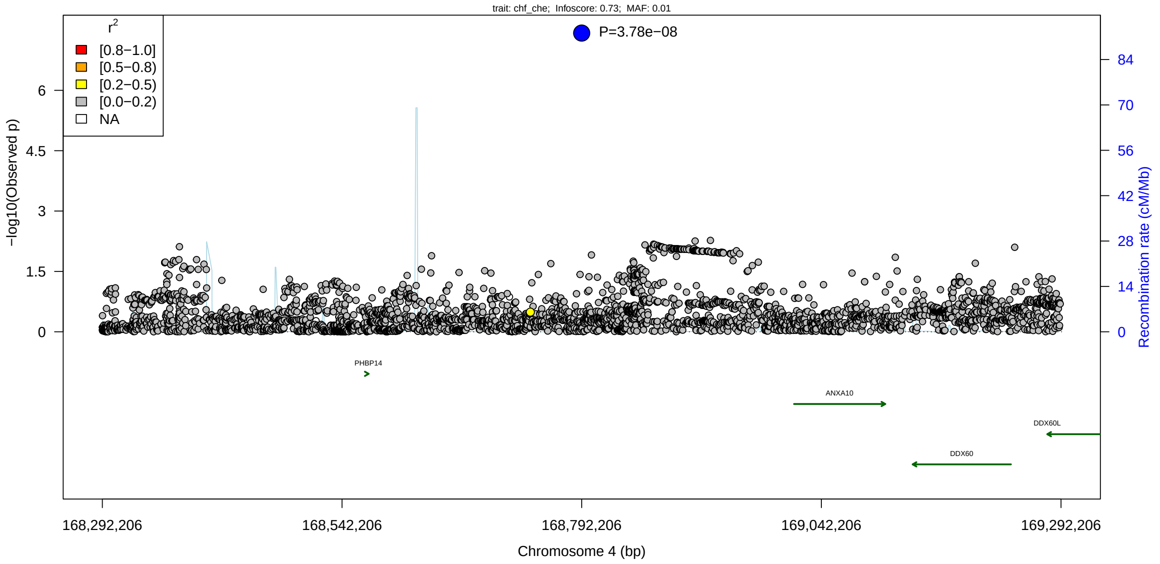

Unsupported Region U3: rs77010382

trait: def_chf; Infoscore: 0.82; MAF: 0.01

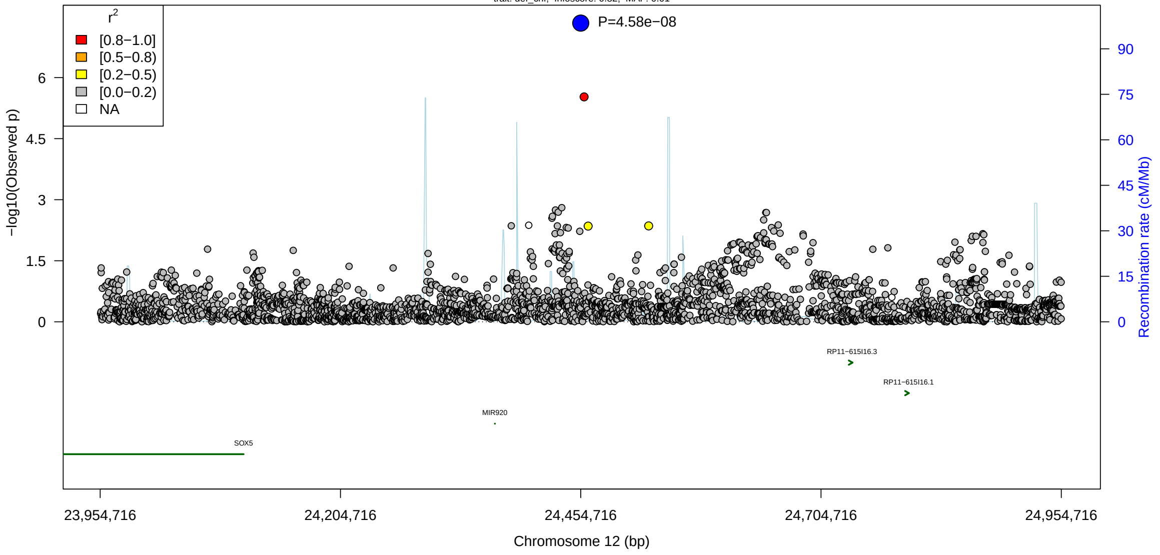

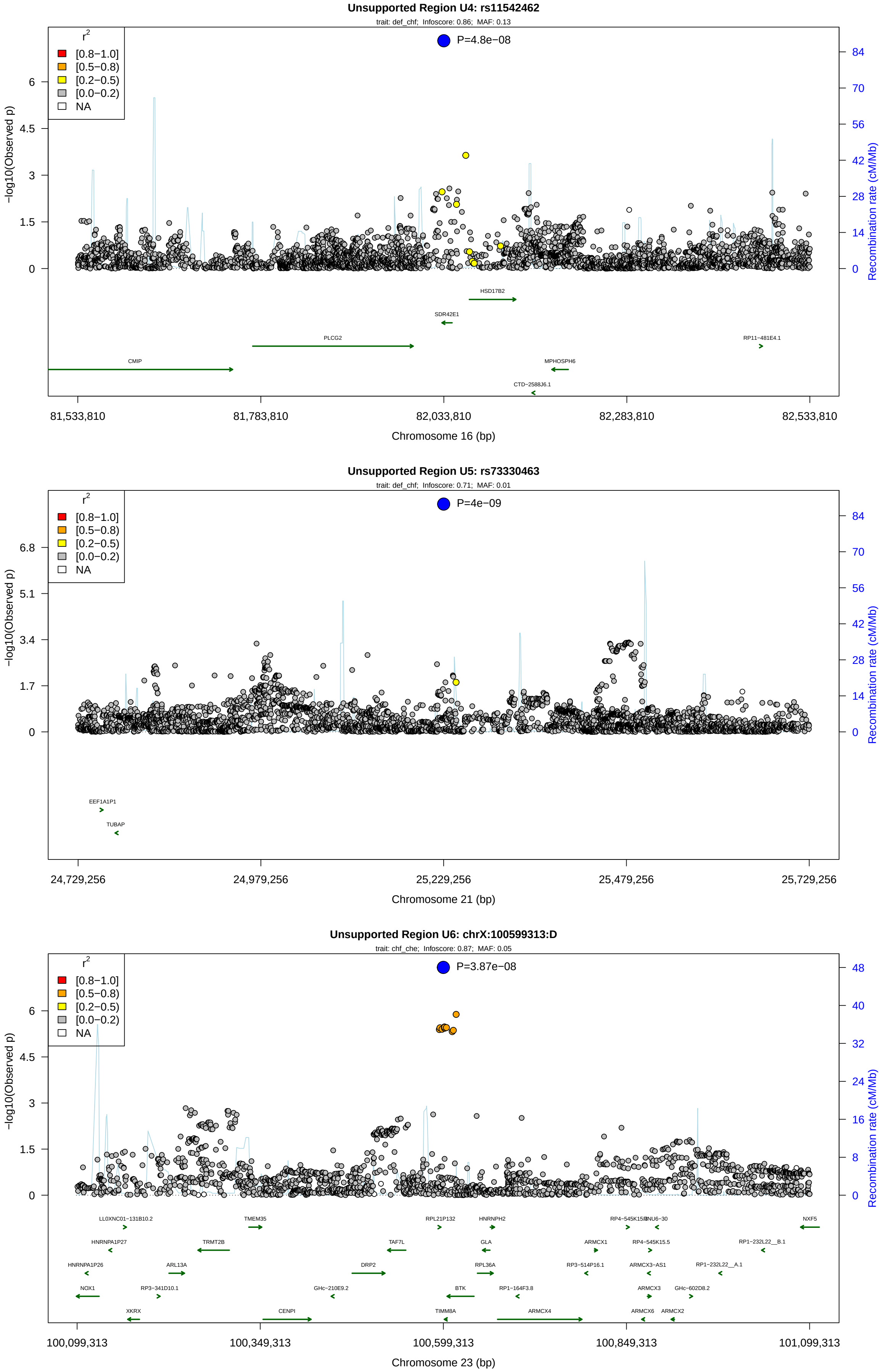

Sup. Fig. S4: Regional association plots of loci with genome-wide significant association with zoosterol traits. Only the best-associated trait is shown for each locus. Loci, where no variant besides the index variant reached at least suggestive significance ($p < 10^{-6}$) were classified as unsupported.

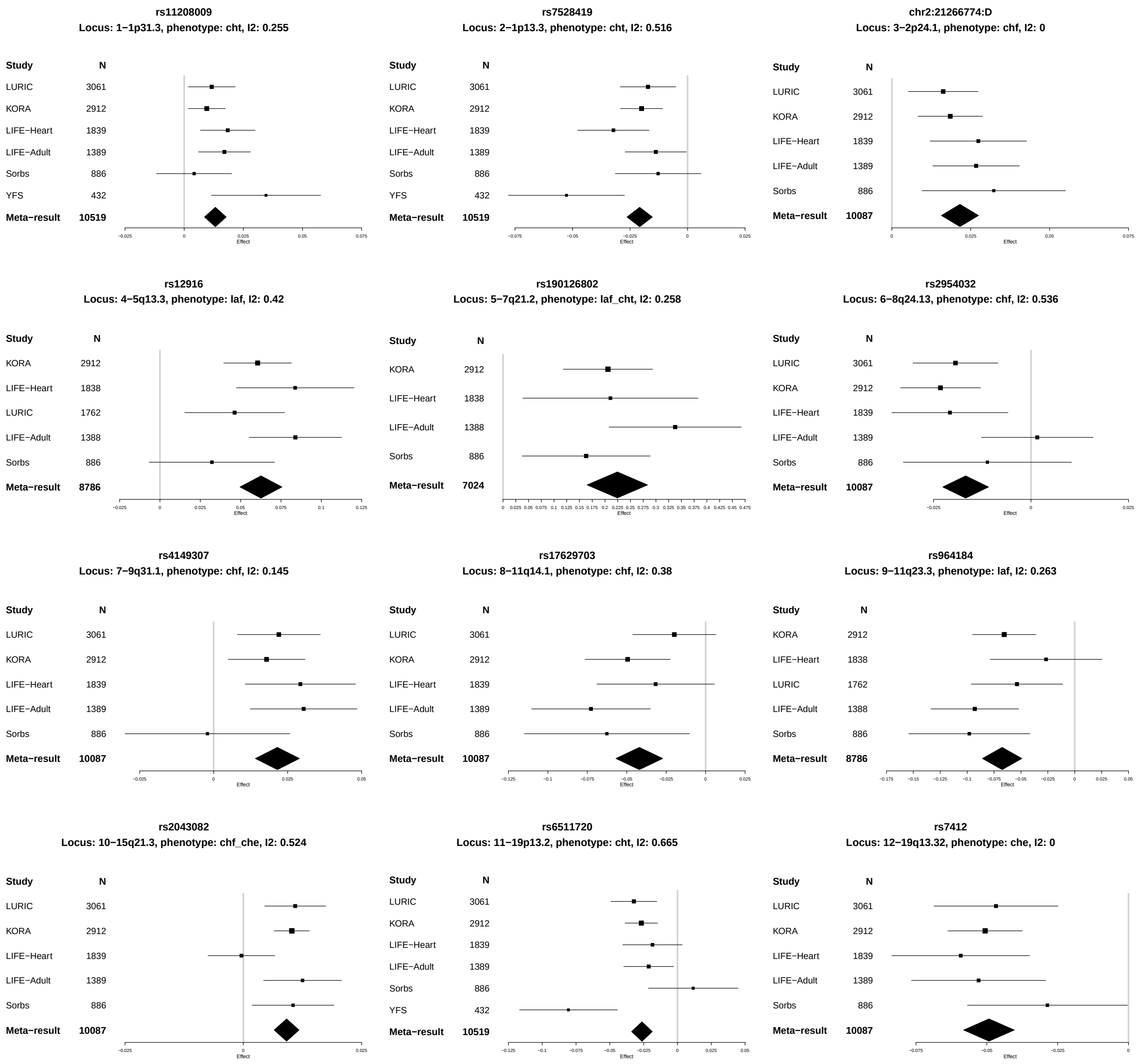

Sup. Fig. S5: Forest plots of supported genome-wide significant loci. Shown are single study effect estimates and corresponding 95% confidence intervals. Meta-results were calculated under a fixed-effect model, weighting studies according to their inverse variance. For each locus, data is shown for the best-associated phenotype. Phenotype abbreviations: laf - free lanosterol, def - free desmosterol, dee - esterified desmosterol, det - total desmosterol, chf - free cholesterol, che - esterified cholesterol, cht - total cholesterol. Ratio traits are indicated with an underscore.

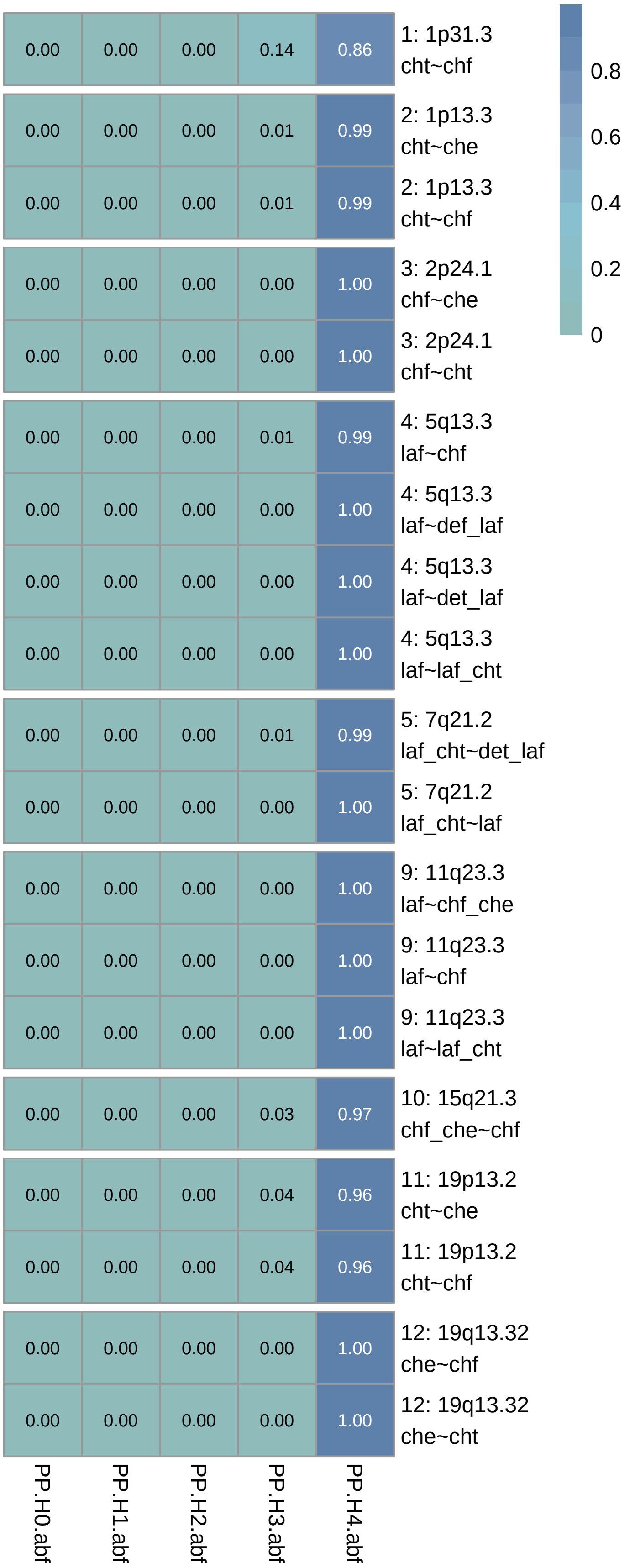

Sup. Fig. S6: Colocalization of traits with genome-wide significant association for loci with multiple associated traits. The analysed pair of traits is separated by a tilde. Phenotype abbreviations: laf - free lanosterol, def - free desmosterol, dee - esterified desmosterol, det - total desmosterol, chf - free cholesterol, che - esterified cholesterol, cht - total cholesterol. Ratio traits are indicated with an underscore.

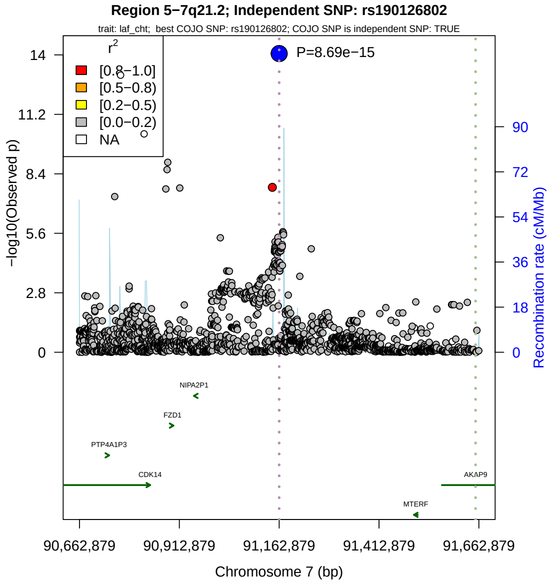
Region 5−7q21.2; Independent SNP: rs190126802
trait: laf_cht; best COJO SNP: rs190126802; COJO SNP is independent SNP: TRUE
P=8.69e−15
r2
[0.8−1.0]
[0.5−0.8]
[0.2−0.5]
[0.0−0.2]
NA
−log10(Observed p)
Recombination rate (cM/Mb)
Chromosome 7 (bp)
90,662,879
90,912,879
91,162,879
91,412,879
91,662,879
NIPA2P1
FZD1
PTP4A1P3
CDK14
AKAP9
MTERF

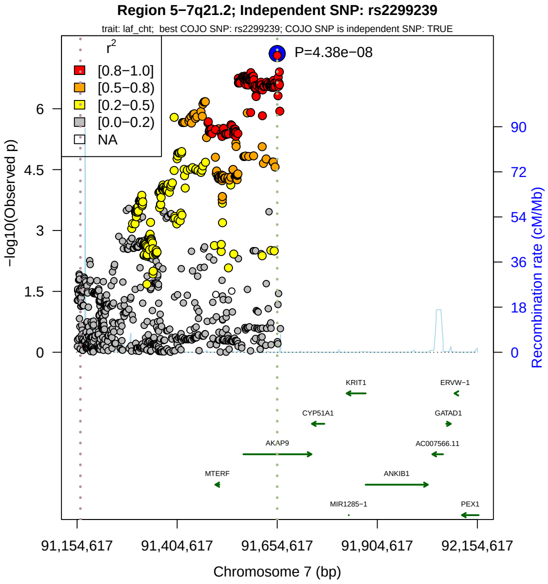
Region 5−7q21.2; Independent SNP: rs2299239
trait: laf_cht; best COJO SNP: rs2299239; COJO SNP is independent SNP: TRUE
P=4.38e−08
r2
[0.8−1.0]
[0.5−0.8]
[0.2−0.5]
[0.0−0.2]
NA
−log10(Observed p)
Recombination rate (cM/Mb)
Chromosome 7 (bp)
91,154,617
91,404,617
91,654,617
91,904,617
92,154,617
KRIT1
ERVW−1
CYP51A1
GATAD1
AKAP9
AC007566.11
MTERF
ANKIB1
MIR1285−1
PEX1

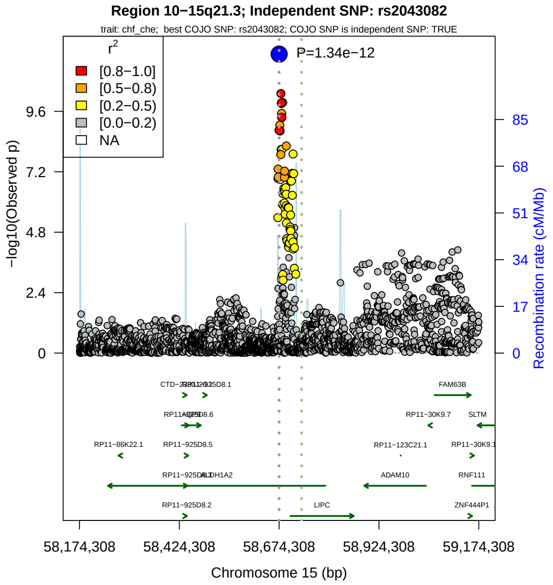
Region 10−15q21.3; Independent SNP: rs2043082
trait: chf_che; best COJO SNP: rs2043082; COJO SNP is independent SNP: TRUE
P=1.34e−12
r2
[0.8−1.0]
[0.5−0.8]
[0.2−0.5]
[0.0−0.2]
NA
−log10(Observed p)
Recombination rate (cM/Mb)
Chromosome 15 (bp)
58,174,308
58,424,308
58,674,308
58,924,308
59,174,308
FAM63B
RP11−30K9.7
SLTM
RP11−86K22.1
RP11−925D8.5
RP11−123C21.1
RP11−30K9.1
ADAM10
RNF111
RP11−925D8.2
LIPC
ZNF444P1

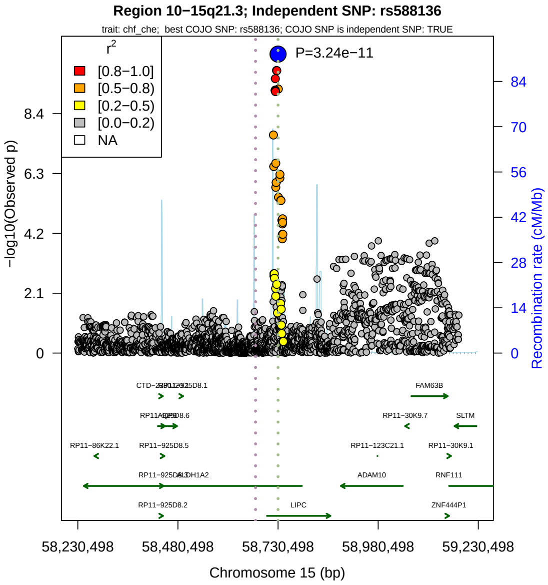
Region 10−15q21.3; Independent SNP: rs588136
trait: chf_che; best COJO SNP: rs588136; COJO SNP is independent SNP: TRUE
P=3.24e−11
r2
[0.8−1.0]
[0.5−0.8]
[0.2−0.5]
[0.0−0.2]
NA
−log10(Observed p)
Recombination rate (cM/Mb)
Chromosome 15 (bp)
58,230,498
58,480,498
58,730,498
58,980,498
59,230,498
FAM63B
RP11−30K9.7
SLTM
RP11−86K22.1
RP11−925D8.5
RP11−123C21.1
RP11−30K9.1
ADAM10
RNF111
RP11−925D8.2
LIPC
ZNF444P1

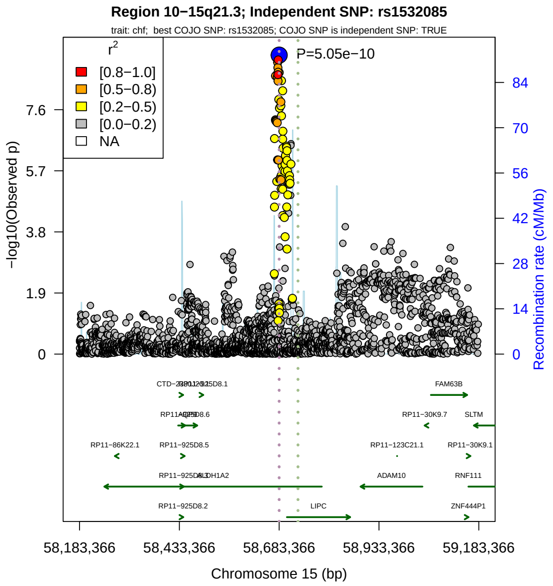
Region 10−15q21.3; Independent SNP: rs1532085
trait: chf; best COJO SNP: rs1532085; COJO SNP is independent SNP: TRUE
P=5.05e−10
r2
[0.8−1.0]
[0.5−0.8]
[0.2−0.5]
[0.0−0.2]
NA
−log10(Observed p)
Recombination rate (cM/Mb)
Chromosome 15 (bp)
58,183,366
58,433,366
58,683,366
58,933,366
59,183,366
FAM63B
RP11−30K9.7
SLTM
RP11−86K22.1
RP11−925D8.5
RP11−123C21.1
RP11−30K9.1
ADAM10
RNF111
RP11−925D8.2
LIPC
ZNF444P1

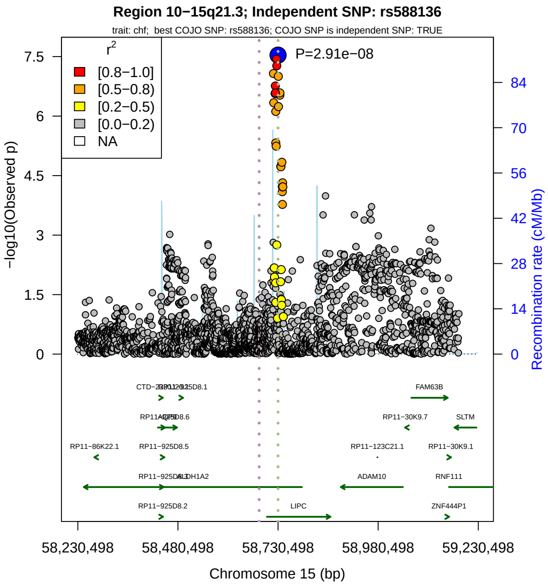
Region 10−15q21.3; Independent SNP: rs588136
trait: chf; best COJO SNP: rs588136; COJO SNP is independent SNP: TRUE
P=2.91e−08
r2
[0.8−1.0]
[0.5−0.8]
[0.2−0.5]
[0.0−0.2]
NA
−log10(Observed p)
Recombination rate (cM/Mb)
Chromosome 15 (bp)
58,230,498
58,480,498
58,730,498
58,980,498
59,230,498
FAM63B
RP11−30K9.7
SLTM
RP11−86K22.1
RP11−925D8.5
RP11−123C21.1
RP11−30K9.1
ADAM10
RNF111
RP11−925D8.2
LIPC
ZNF444P1

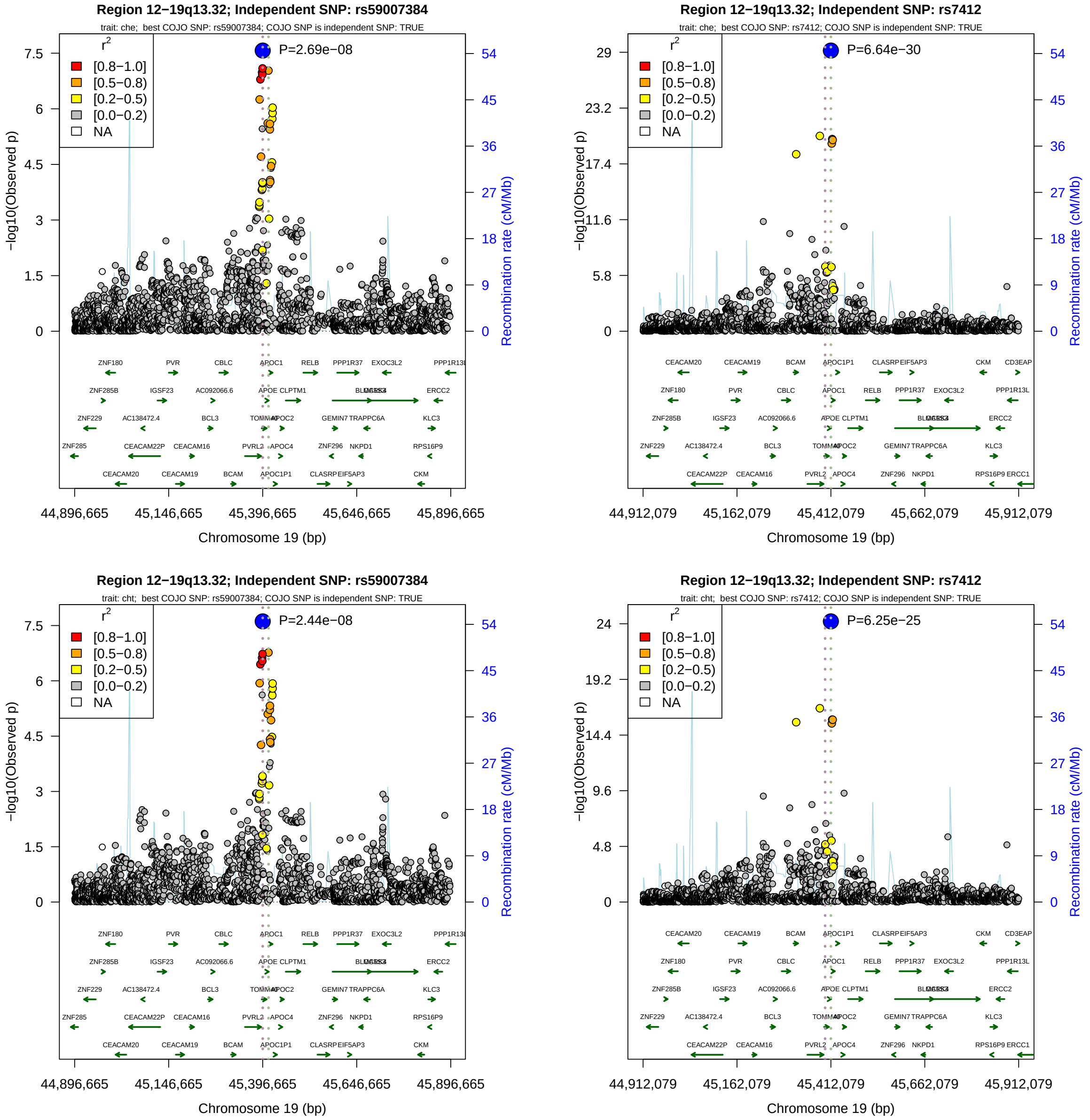

Sup. Fig. S7: Regional-association plots based on conditional statistics for loci with multiple independent variants. Conditional statistics for the best associated trait were obtained for the specified SNP by conditioning on all other independent variants at the respective locus. This procedure was iterated for each phenotype where multiple independent variants were found. Positions of independent variants are indicated by dotted lines. The variant with the strongest association after conditioning is shown as 'best COJO SNP'. If this variant is identical to the independent variant, the entry 'COJO SNP is independent SNP' is set to TRUE.

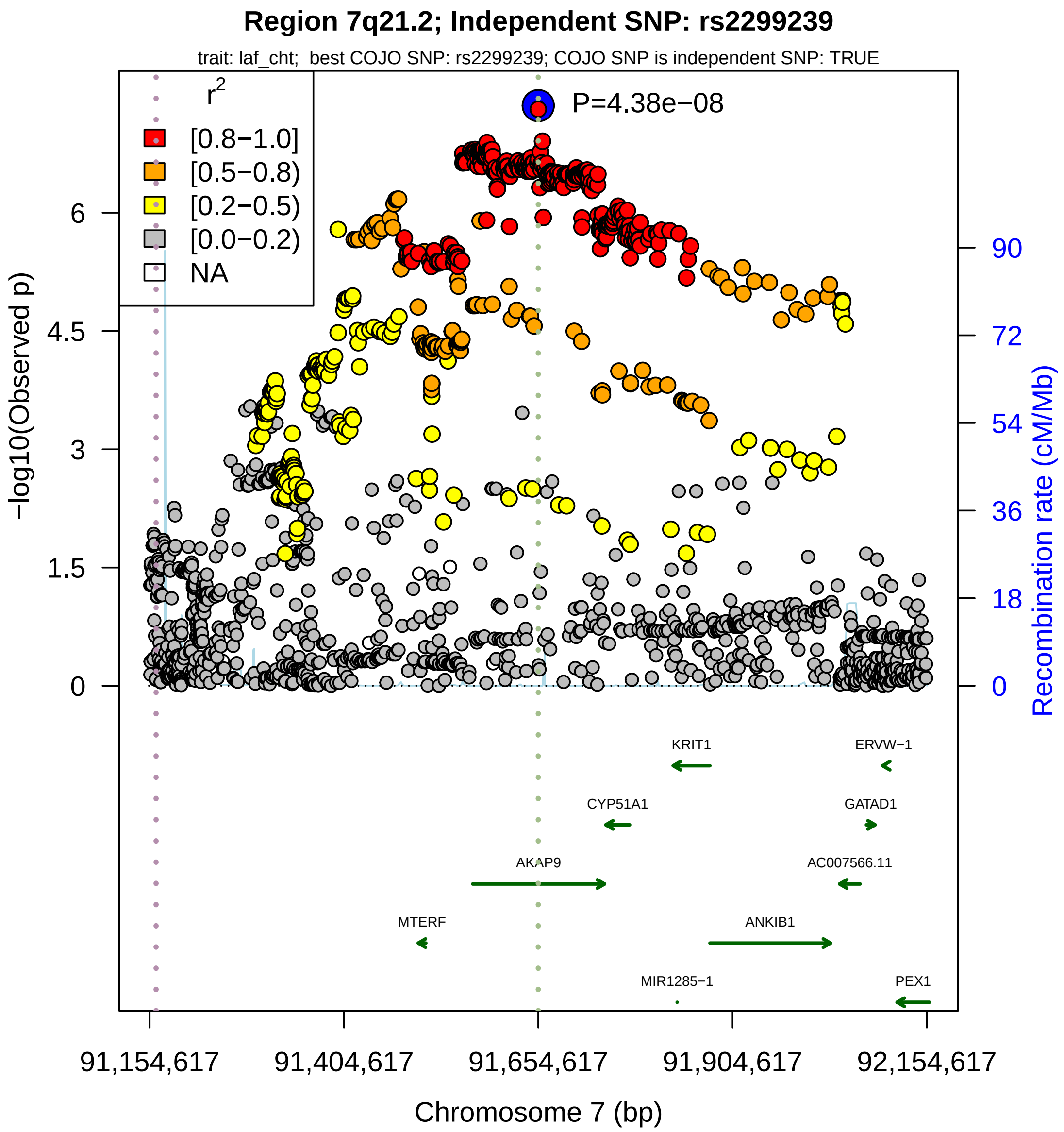

Sup. Fig. S8: Regional-association plot based on conditional statistics for the secondary independent variant rs2299239 found at locus 7q21.2. The original locus was extended 500 kb downstream before conditioning on the index variant rs190126802.

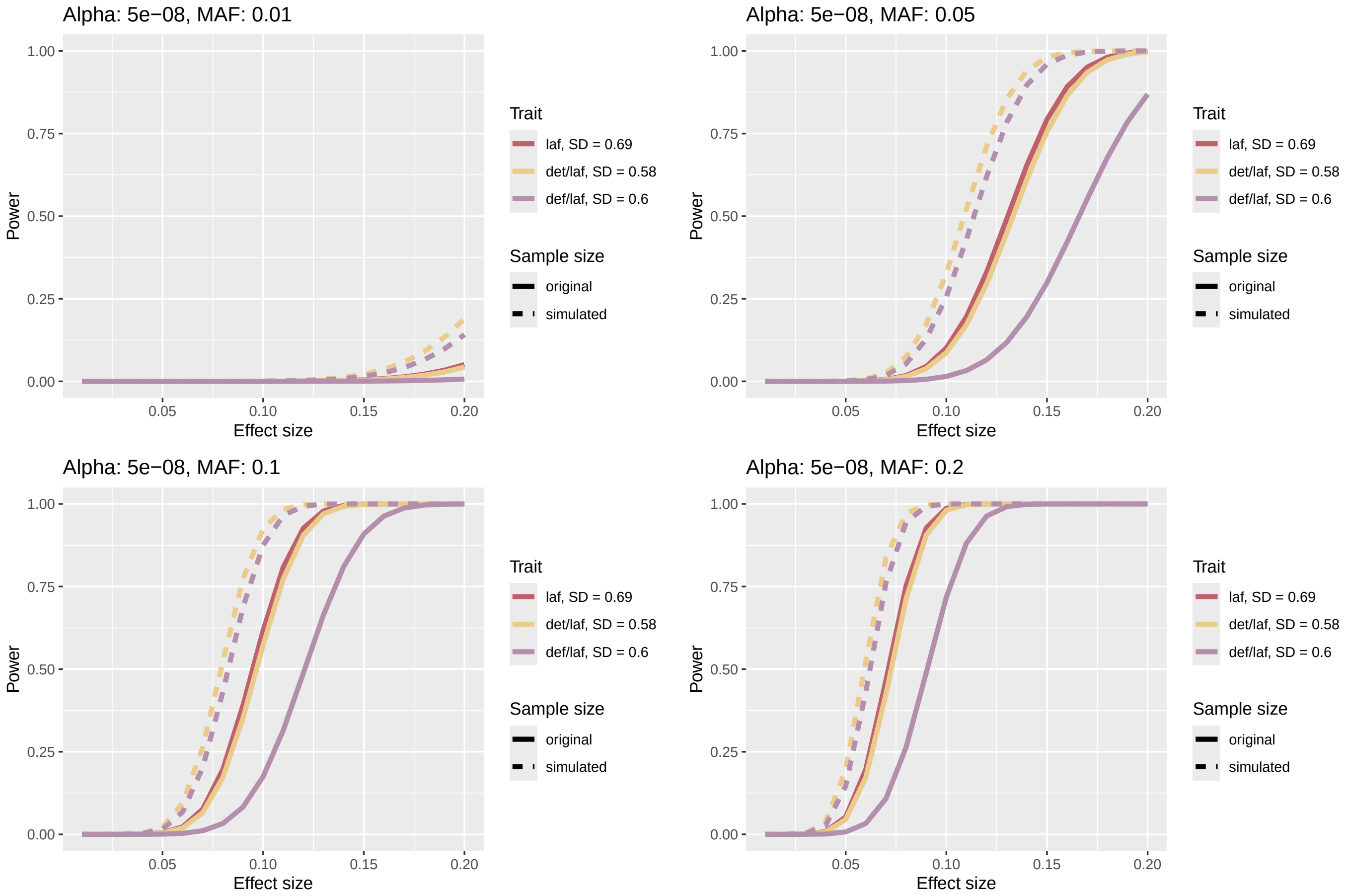

Sup. Fig. S9: Power analysis for lanosterol and desmosterol to lanosterol ratios for a range of minor allele frequencies. Power was calculated for free lanosterol (laf), total desmosterol to lanosterol ratio (det/laf), and free desmosterol to lanosterol ratio (def/laf). Original sample sizes (laf: 8786, det/laf: 5873, def/laf: 4111) were used. We also simulated power for desmosterol ratios, when sample size increased to that of free lanosterol. The x-axis shows the effect size and the y-axis the power. We assumed an additive genetic model. Standard deviation (SD) of traits was calculated in LIFE-Heart.
